# The Concept of Scaffold-Guided Bone Regeneration for the Treatment of Long Bone Defects: Current Clinical Application and Future Perspective

**DOI:** 10.3390/jfb14070341

**Published:** 2023-06-27

**Authors:** Markus Laubach, Frank Hildebrand, Sinduja Suresh, Michael Wagels, Philipp Kobbe, Fabian Gilbert, Ulrich Kneser, Boris M. Holzapfel, Dietmar W. Hutmacher

**Affiliations:** 1 Australian Research Council (ARC) Training Centre for Multiscale 3D Imaging, Modelling and Manufacturing (M3D Innovation), Queensland University of Technology, Brisbane, QLD 4000, Australia; 2 Centre for Biomedical Technologies, School of Mechanical, Medical and Process Engineering, Queensland University of Technology, Brisbane, QLD 4059, Australia; 3 Department of Orthopaedics and Trauma Surgery, Musculoskeletal University Center Munich (MUM), LMU University Hospital, LMU Munich, Marchioninistraße 15, 81377 Munich, Germany; 4 Department of Orthopaedics, Trauma and Reconstructive Surgery, RWTH Aachen University Hospital, Pauwelsstraße 30, 52074 Aachen, Germany; 5 Department of Plastic Surgery, Princess Alexandra Hospital, Woolloongabba, QLD 4102, Australia; michael.wagels@health.qld.gov.au; 6 The Herston Biofabrication Institute, The University of Queensland, Herston, QLD 4006, Australia; 7 Southside Clinical Division, School of Medicine, University of Queensland, Woolloongabba, QLD 4102, Australia; 8 Department of Plastic and Reconstructive Surgery, Queensland Children’s Hospital, South Brisbane, QLD 4101, Australia; 9 The Australian Centre for Complex Integrated Surgical Solutions, Woolloongabba, QLD 4102, Australia; 10 Department of Hand, Plastic and Reconstructive Surgery, Microsurgery, Burn Center, BG Trauma Center Ludwigshafen, University of Heidelberg, 67071 Ludwigshafen, Germany; 11 Max Planck Queensland Centre (MPQC) for the Materials Science of Extracellular Matrices, Queensland University of Technology, Brisbane, QLD 4000, Australia; 12 ARC Training Centre for Cell and Tissue Engineering Technologies (CTET), Queensland University of Technology (QUT), Brisbane, QLD 4000, Australia

**Keywords:** large volume bone defects, scaffold, bone regeneration, 3D printing, additive manufacturing, titanium, composites, aliphatic polyester, polycaprolactone

## Abstract

The treatment of bone defects remains a challenging clinical problem with high reintervention rates, morbidity, and resulting significant healthcare costs. Surgical techniques are constantly evolving, but outcomes can be influenced by several parameters, including the patient’s age, comorbidities, systemic disorders, the anatomical location of the defect, and the surgeon’s preference and experience. The most used therapeutic modalities for the regeneration of long bone defects include distraction osteogenesis (bone transport), free vascularized fibular grafts, the Masquelet technique, allograft, and (arthroplasty with) mega-prostheses. Over the past 25 years, three-dimensional (3D) printing, a breakthrough layer-by-layer manufacturing technology that produces final parts directly from 3D model data, has taken off and transformed the treatment of bone defects by enabling personalized therapies with highly porous 3D-printed implants tailored to the patient. Therefore, to reduce the morbidities and complications associated with current treatment regimens, efforts have been made in translational research toward 3D-printed scaffolds to facilitate bone regeneration. Three-dimensional printed scaffolds should not only provide osteoconductive surfaces for cell attachment and subsequent bone formation but also provide physical support and containment of bone graft material during the regeneration process, enhancing bone ingrowth, while simultaneously, orthopaedic implants supply mechanical strength with rigid, stable external and/or internal fixation. In this perspective review, we focus on elaborating on the history of bone defect treatment methods and assessing current treatment approaches as well as recent developments, including existing evidence on the advantages and disadvantages of 3D-printed scaffolds for bone defect regeneration. Furthermore, it is evident that the regulatory framework and organization and financing of evidence-based clinical trials remains very complex, and new challenges for non-biodegradable and biodegradable 3D-printed scaffolds for bone regeneration are emerging that have not yet been sufficiently addressed, such as guideline development for specific surgical indications, clinically feasible design concepts for needed multicentre international preclinical and clinical trials, the current medico-legal status, and reimbursement. These challenges underscore the need for intensive exchange and open and honest debate among leaders in the field. This goal can be addressed in a well-planned and focused stakeholder workshop on the topic of patient-specific 3D-printed scaffolds for long bone defect regeneration, as proposed in this perspective review.

## 1. Introduction

The organ bone has a remarkable ability to regenerate itself after injury; this unique regenerative ability, shared perhaps only with the adult human liver, allows bones to rebuild to a shape, size, and strength essentially the same as before injury [1]. In critical-sized bone defects, which affect millions of people worldwide and are caused by infection, malformation, tumour resection, and traumatic injuries, bone cannot spontaneously self-regenerate [2,3]. Bone defects can be divided into two distinct groups: cavity defects, wherein the loss does not affect limb biomechanics but still interferes with osteosynthesis or endoprosthesis implantation; and segmental defects, wherein normal biomechanics are compromised, and the structural stability of the bone as an organ is at risk [4]. In spite of the progress and optimization of surgical techniques and postoperative care, the treatment outcome of bone defects is not always predictable, and the morbidity should not be underestimated. Henceforth, as evidenced in this review, it remains a challenge for both surgeons and patients [5]. An interdisciplinary approach which is rooted in planning and communication between surgical disciplines, e.g., orthopaedic surgeons, plastic surgeons, and vascular surgeons, to name a few, is necessary to incorporate vigilant patient selection, including host optimization, and preoperative consultation into the treatment process to further improve clinical outcomes [6,7,8,9]. Therefore, the management of segmental defects often requires interdisciplinary patient work-up [9] and multiple surgeries, and most techniques used to reconstruct limbs are associated with long treatment times, unpredictable bone union rates, or methods that are poorly tolerated by patients [10,11,12]. Notably, in addition to the life-threatening challenges in the acute phase of bone loss, patients with severe limb injuries suffer long-term consequences in their professional and social lives as well. Functional and cognitive impairments, psychological condition, degree of disability and occupational disability determine the long-term course of these patients [13,14].

**Figure 1:**
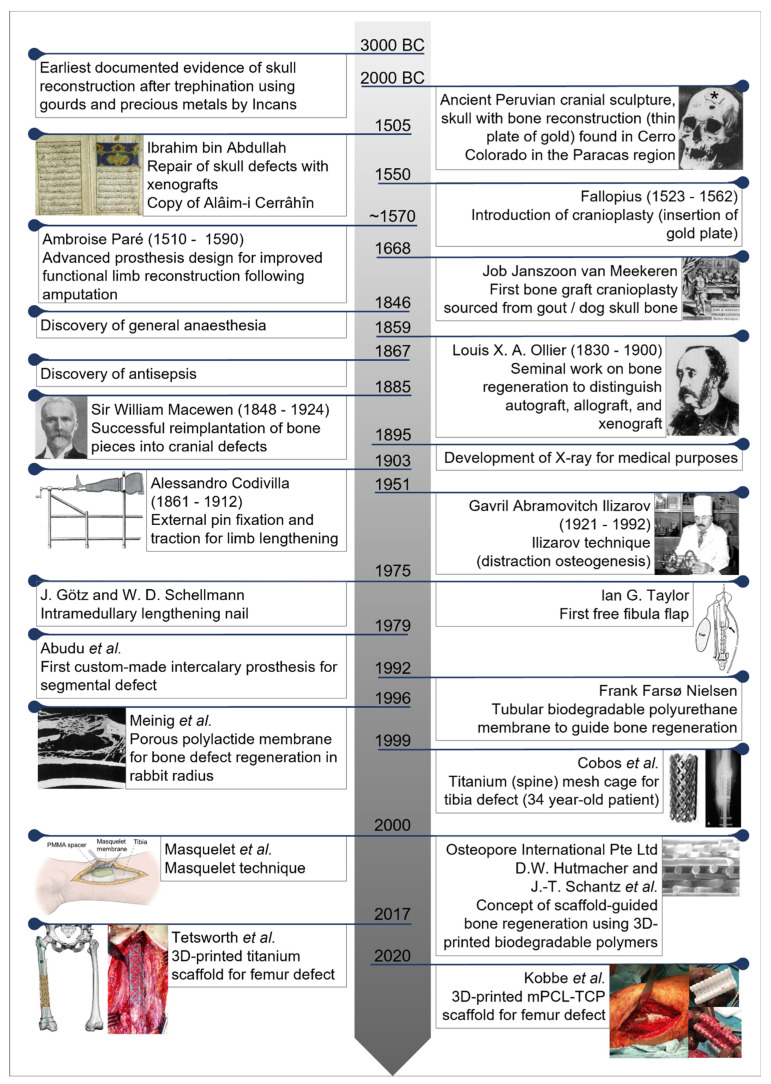
Selection of key historical advancements relevant for bone defect treatment concepts. Original prologue page from the oldest copy of Alâim-i Cerrâhîn [15]. Skull from Cerro Colorado in the Paracas region of Peru: A thin gold plate covers the left forehead (*), from Tello JC: Antiguo Peru: Primera Epoca. Peru, Lima, 1929, p. 148 [16]. Title page of Meekeren’s book ‘Observationes Medico-Chirugicae’ describing the first bone grafting on the skull, from Meekeren JJ: Observationes Medico-Chirugicae. Amsterdam, Ex Officina Henrici & Vidnae Theodori Boom, 1682 [17]). Free fibula flap by Taylor (1974): Free soft tissue flap attached to the anterior tibial artery with anastomosis of the fibular graft (arrow) to the peroneal artery; image reprinted from Ref. [18], with permission from Wolters Kluwer Health, Inc. Copy of Alâim-i Cerrâhîn reprinted with permission from Ref. [19]. Photo of Louis X.A. Ollier (1830–1900) reprinted from Ref. [20], with permission from Wolters Kluwer Health, Inc. (Philadelphia, PA, USA). Photo of Sir William Macewen (1848–1924) reprinted from Ref. [20], with permission from Wolters Kluwer Health, Inc. Image of porous polylactide membrane (Meinig et al.) reprinted from Ref. [21], with permission from Wolters Kluwer Health, Inc. Image of first titanium (spinal) mesh cage for tibia defect (Cobos et al.) reprinted from Ref [22], with permission from John Wiley and Sons. First 3D-printed mPCL-TCP scaffold for femur defect adapted from Ref. [23]. Apparatus Codivilla reprinted from Ref. [24], with permission from Wolters Kluwer Health, Inc. Prof. Gavril Abramovitch Ilizarov (1921–1992) in his study reprinted from Ref. [25]. Masquelet technique image adapted from Ref. [26]. Image next to ‘Osteopore International Pte Ltd.’ reprinted from Ref. [27]. Figure adapted from Refs. [20,28,29,30,31].

The main objective of this article is to review and conceptualize the application of the increasingly clinically applied concept of scaffold-guided bone regeneration (SGBR) to the extremely challenging problem of segmental long bone loss. To achieve this, the main aim is to evaluate the clinical utility of patient-specific 3D-printed scaffolds and place them in the historical context (Figure 1). Furthermore, it provides a perspective of current treatment methods to guide the reader with a holistic conceptual understanding of SGBR in its entirety.

### Central Historical Events

This review is primarily concerned with lower limb long bone defect treatment. From a historical perspective, however, the modern treatment of long bone defects is based to a considerable degree on the insights gained from the (surgical) management of cranial bone defects. Although this review focuses primarily on the appendicular skeleton, it is therefore useful to review the historical discussion of the knowledge gained from the treatment of cranial bone defects that is relevant to the current therapy of long bone defects. Surgical treatment of bone defects dates back to at least 3000 BC, where archaeological evidence suggests that the Incas, in what is now Peru, reconstructed trepanation of the head on living subjects using shells, gourds, and silver or gold plates [20,29,32]. In Europe, reconstruction of cranial bone defects was introduced by Fallopius (1523–1562), who advised replacing the bone if the dura was not injured; if the dura was injured, he suggested the bone should be removed and a gold plate inserted. In 1668, the Dutch surgeon Job Janszoon van Meekeren reported, although notably did not perform himself [20], the transplantation of a dog bone into the skull defect of a Russian man [17]. This is the first known bone graft [28], which was to be removed by order of the church [33], but as the bone had already grown into the soldier’s skull, the graft could no longer be removed [34].

The means of amputation combined with a well-fitting prosthesis was the earliest form of functional reconstruction of the lower limbs, with the French barber and surgeon Ambroise Paré being one of the pioneers of this philosophy in the 16th century [35]. In 1885, Macewen, who is considered the father of modern bone grafting, reported the successful reimplantation of bone fragments into skull defects, popularizing the bone grafting procedure [30]. Experiments with synthetic materials began in the 19th century with materials such as wood or marble, but the first promising synthetic material was plaster of Paris consisting of calcium sulphate in 1892 [34,36,37]. However, improved surgical treatment options for bone defect regeneration were not observed until the profound discoveries of general anaesthesia (1846), antisepsis (1867), and X-ray technology (1895), allowing for overall improved treatment outcomes [38,39,40,41].

Using autologous bone graft (ABG) in bone regeneration concepts was pioneered by Havers (1692), Ollier (1867), and Senn (1889) [42]. In particular, Ollier was one of the first to distinguish and describe the terms autograft, allograft, and xenograft by performing bone grafting experiments for bone regeneration in animals [30]. The destructive nature of warfare in the 20th century and increasing violence of civilian trauma in the modern era further spurred the search for alternative bone substitutes such as metals and plastics and alternative methods such as limb lengthening procedures, as well as improved microvascular instruments and skills to treat long bone defects [43]. Notably, titanium was first used by Simpson (1965) and has continued to be used for cranioplasty [44,45] as well as in the reconstruction of segmental long bone defects. Limb lengthening as an ancient approach to defect filling can be traced back to the nineteenth century [46]. The first attempts at limb lengthening were performed as single-stage lengthening osteotomies by pioneers such as von Langenbeck (1869), Hopkins and Penrose (1889), and von Eiselsberg (1897) [46,47].

Modern limb lengthening techniques began with the work of Italian surgeon Alessandro Codivilla in Bologna, Italy, who used external pins and traction for bone lengthening, reporting his work in 1903 [24,48,49]. Since then, the orthopaedic community has made tremendous strides in performing successful lengthening surgeries, with contributions from one of the most prominent leaders in the field: Prof. Gavril Ilizarov [46]. Ilizarov, working in Kurgan, Siberia, first introduced the technique of a circular external skeletal fixation system attached to bone with tensioned transfixation wires to treat a long bone defect caused by tuberculosis in 1951, successfully combining the surgical principles of limb lengthening with the biology of distraction histiogenesis [50,51]. Moreover, an improved understanding of bone vascularity in the 20th century led to vascularized bone transfers [52,53], with the first free fibula flap being performed by Taylor in 1974, particularly with the aid of the operating microscope, for a 14 cm tibial defect [18]. The first publication on an intramedullary lengthening nail dates back to 1975, whereby lengthening was achieved by an external transcutaneous connection, i.e., a hydraulic pump connected to the nail tip, driving the telescopic mechanism of the nail [54]. Since then, intramedullary bone lengthening (using intramedullary nails) has undergone continuous technical development and can lead to a widely satisfactory clinical outcome in a carefully selected group of patients with long bone defects [55,56,57,58,59].

However, in today’s world, the number of cases of (post-traumatic) bone defects is increasing dramatically [60,61], imposing an enormous socioeconomic burden [62,63,64], especially as we live in the “age of global aging” in which by 2050 about one-fifth of the world’s population will be 60 years and older due to increased life expectancies [65]. Thus, the need for advanced and sophisticated treatment alternatives for long bone defects is increasing.

## 2. Bone Defects: Current Treatment Methods

**Figure 2:**
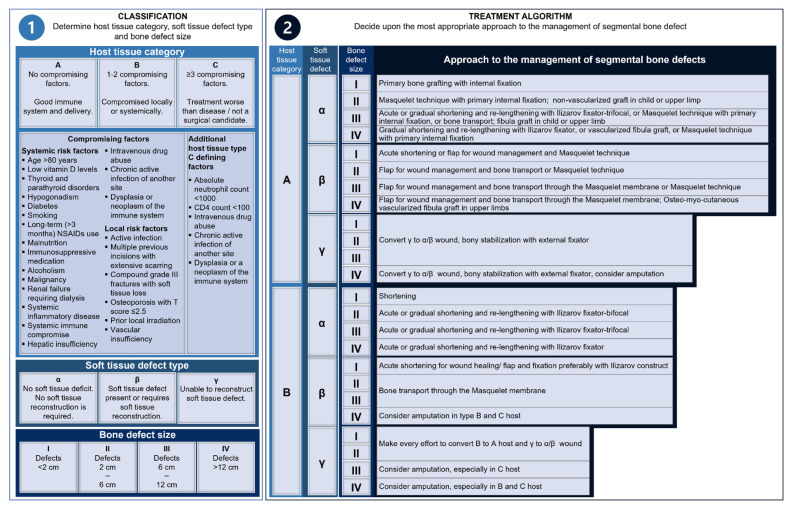
Management algorithm for segmental bone defects. Please note that the host status can be graded using the McPherson system, which is derived from the Cierny and Mader classification [75], and divides the patients in three host tissue categories (A, B, or C) depending on the number of comorbidities [76,77]. Adapted from Ref. [6].

There are a multitude of factors that influence the outcome of treatments for bone defects, and these should be considered when selecting the most appropriate surgical intervention and postoperative care for each patient. A variety of options have been described for the treatment of bone defects depending on the patient health record, the location of the defect, and the surgeon’s preference and experience, and several algorithms have been proposed in the past [66]. Notably, based on three main components, namely the size of the defect, the ability to reconstruct the soft tissue envelope, and the overall host (patient) status, an algorithmic approach for segmental bone defects has been proposed by Ferreira and Tanwar [6] and adapted by our group (Figure 2). Limb reconstruction primarily starts with the focus of the host (tissue), followed by soft tissue reconstruction, and finally, the bony reconstruction. The bone defects are mainly treated with (autologous, allograft, synthetic) bone grafts to fill the defect side and regenerate the bone. For historical and biological reasons, ABG is considered the “gold standard” among graft materials. ABG is the only graft material that is osteogenic and fulfils all three components of the regeneration triad (osteogenesis, osteoinduction, and osteoconduction) [67]. The increase in frequency and volume of bone grafts used in orthopaedic surgery, however, led to the requirement to expand the autograft. Therefore, in orthopaedic clinics, allografts, xenografts, and synthetic bone graft substitutes are used as supplementary graft material to treat bone defects [68], but they face critical constraints, including supply shortages, donor site morbidity, disease transmission, immune rejection, and high costs [69,70]. These limitations and the advantages of ABG including short healing times, favourable bone quality, lower material costs, no risk of disease transmission or antigenicity, and predictability ABGs will remain key components in bone defect treatment strategies [67,71,72]. However, ABG used to regenerate larger bone defects (>3–5 cm) is associated with an increased risk of graft resorption [73] and biomechanical weakness of the reconstructed segment [74].

Alternative treatment approaches, historically and to date, for treating larger long bone defects are based on vascularized free bone transfer [78,79,80], the Ilizarov intercalary bone transport method (distraction osteogenesis) [81], massive bone grafts [82], amputation [83], or (arthroplasty with) mega-prosthesis [84]. However, these methods are technically demanding and associated with high complication rates [85,86]. While we propose a management algorithm (Figure 2) that can support rational decision-making of the interdisciplinary clinical team, it is important to recognize that this algorithm is not dogmatic and that the most appropriate procedure or combination of treatment methods for reconstruction of long bone defects needs to be determined individually for each patient [6]. Notably, the current literature indicates that mainly patients under 50 years are suffering from massive long-bone defects [23,87,88], and this group’s cost-associated benefits from limb salvage are significant in the long term (≥7 years) when contrasted with primary limb amputation [89]. However, the preservation of limbs is challenging in critical-sized bone defects [90]. In particular, bone transport, which notably offers the unique opportunity to simultaneously address deformity and limb length discrepancy, is characterized by a long treatment time (on average >12 months) with (external) fixation, which is associated with considerable discomfort for the patient and requires high patient compliance [91,92]. A protracted course of treatment is often undertaken, influencing the patient’s quality of life, possibly involving frequent revision surgeries and failure of revision procedures, posing major surgical and socio-economic challenges [12,93]. Moreover, the long duration of treatment is less attractive, especially for socially disadvantaged persons, those with mental illnesses, and people with poor support environments [94].

To address these limitations of conventional long bone defect treatment methods, the Masquelet technique, first described by Alain-Charles Masquelet, has gained popularity as an alternative approach in cases of large, long bone defects [95,96,97,98,99]. Following an osseous and soft tissue debridement, the first step of the Masquelet technique is to use a polymethyl methacrylate (PMMA) spacer loaded with antibiotics to span the bone defect. This cement spacer has been shown to stimulate the formation of an biologically active pseudo-membrane (Masquelet membrane) around it which contains osteoinductive growth factors relevant for bone healing [100]. In the second phase, autologous morselised bone grafts are typically inserted into the Masquelet membrane, which can be at least partially augmented with alternative bone grafts (allografts, synthetics) so that even massive defects can be treated with the Masquelet technique [95,101]. Thus, not only septic but, importantly, also aseptic (segmental) defects of long bones can be managed by this two-stage Masquelet technique [99]. However, despite remarkable clinical success rates, the achievement of bone regeneration over the entire defect distance depends on many factors, such as the anatomical region, the stability of the osteosynthesis, and the soft tissue coverage, which is why several additional procedures are often necessary and a failure rate of 10% to 15% is reported for the Masquelet technique [102,103,104]. Of note, contradictory results are published discussing osteoinductive or osteoconductive (bone graft) material best used inside the Masquelet membrane [103,105]. In summary, these significant clinical challenges of historical and contemporary methods for the treatment of bone defects, which require the generation of new bone as opposed to ‘more simple’ fracture repair (see Table 1—Definition 1), have led the Tissue Engineering and Regenerative Medicine (TE&RM) research community to develop concepts for non-biodegradable and biodegradable implants for the regeneration of (long bone) tissue.

Definition 2

## 3. An alternative Concept: ‘Guided Tissue Engineering’

**Figure 3:**
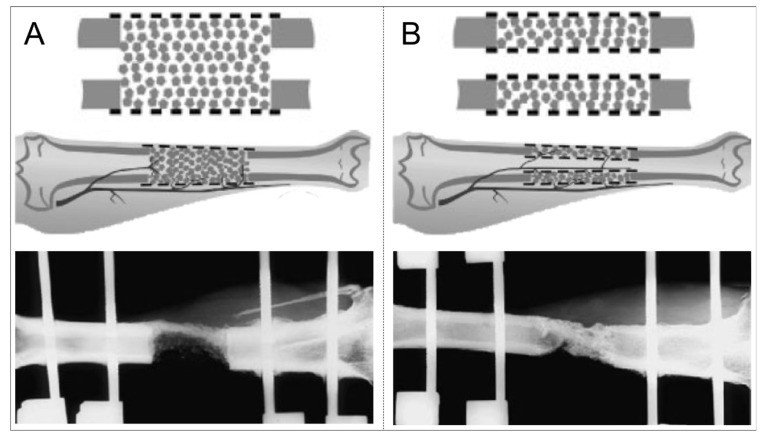
Schematic representation of Gugala and Gogolewski [120]’s study design using the polylactide membranes as well as radiographs of the defects sixteen weeks after sheep tibia segmental defect surgery. The single perforated membrane loaded with cancellous bone graft resulted in significant resorption of the autograft in the antero-medial region of the grafted defects (**A**). The defects with grafted double mesh membrane (“tube-in-tube” membrane implants) exhibited greater bone formation compared to the defects with single mesh membrane, which may be attributed to more uniform reconstitution of the graft (**B**). Adapted from Ref. [22], reproduced with permission from John Wiley & Sons, Inc.

Based on work on biodegradable polymers by underlying macromolecular degradation [27,31,116,117], an interdisciplinary team at the AO (Association of the Study of Internal Fixation) Foundation (Davos, Switzerland) used a biodegradable polyurethane membrane shaped as a tube to bridge a segmental defect. The membrane was fixed via a press fit overlap on the bone ends by 3 mm in rabbits and resulted in callus formation external to the membrane fusing the bone defect [118]. Therefore, it became evident that membranes can avoid interposing muscle and fibrous soft tissue during bone defect healing as well as serve as a “scaffold” in the sense of ‘guided tissue regeneration’ for bone regeneration, but this requires a porous property to achieve bone formation within the membrane and, thus, in the area of the bone defect [118]. Moreover, reported in 1996, tubular porous membranes made of aliphatic polyesters (polylactide) were used as this biodegradable polymer had a track record in Food and Drug Administration (FDA)-approved medical devices. The treatment of segmental bone defects facilitated osseous regeneration across a diaphyseal defect in rabbits and paved the way for subsequent large animal studies [21,119]. The subsequent sheep study of a segmental 4 cm tibial defect stabilized with bilateral external fixator resulted in two key findings: (1) defect healing could not be achieved without additional ABG application, and polylactide micro-porous membranes (pores 50–70 µm in size) without additional larger pores were not suitable to guide bone regeneration [120]; (2) the polylactide micro-porous mesh membranes with additional laser-perforation (adding additional larger pores), in single or double (cylinder-within-cylinder) configurations (mesh membranes had laser created perforations with a size of 800–900 µm), showed that when used with ABG, the double configuration was associated with significantly more bone formation, but notably, both configurations resulted in bone regeneration [120] (Figure 3).

### 3.1. Preclinical Testing (Spinal) Porous Titanium Mesh Cages for Long Bone Defects

Following these successful proof-of-principle studies, the goal was to test FDA-approved implants with a similar design to repurpose an implant for use in long bone segment defects. Metals have historically been known for their excellent mechanical properties, especially for supporting loads [121]. Metallic biomaterials, including titanium and its alloys, have long been clinically used materials for the fabrication of dense, mesh or porous (3D-printed) implants [69]. FDA approval of titanium mesh cages for the reinforcement of any bone was received in 1990 [5]. Cylindrical titanium mesh cages, although FDA-approved for all bone reconstruction and/or reinforcement, were, until then, developed and exclusively used in the spine for vertebral body replacement and/or interbody fusion. In an attempt to repurpose these titanium implants, critically sized 3 cm segmental femoral defects of canines were reconstructed with a commercially available titanium mesh cage (DePuy Motech, J&J; Warsaw, IN, USA) with diamond-shaped fenestrations [122]. The titanium mesh cages were filled with an allograft composite consisting of crushed fresh-frozen canine cancellous bone and demineralized canine bone matrix, and the defects were stabilized with either an intramedullary titanium nail or a titanium plate and screws [122]. Complete defect bridging was observed in both the cage–nail and cage–plate constructs, resulting in new bone formation within and around the cage and throughout the defect constructs [122].

In conclusion, cylindrical, fenestrated implants in the form of large porous polylactide mesh membranes as well as titanium (spinal) mesh cages could accommodate grafting with cancellous bone graft and facilitate bone regeneration in critical-sized defects in large animal models. The hollow, fenestrated geometry of the polylactide and titanium meshes allowed both the insertion of various biologically active adjuvants consisting of bone grafts or bone substitutes and the diffusion of nutrients as well as the promotion of vascular ingrowth to eventually supplement its biological effectiveness [22].

### 3.2. Clinical Application Cylindrical (Spinal) Titanium Mesh Cages for Long Bone Defects

**Figure 4:**
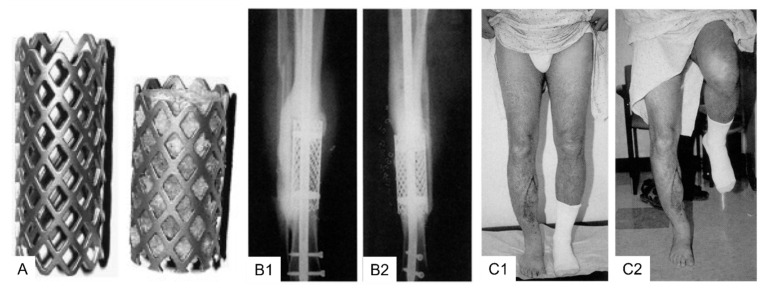
First case report from 1999 by Cobos et al. [123] on the treatment of a patient (34-year-old) with post-traumatic segmental defect of the tibia (8.5 cm) with a cylindrical (spinal) titanium mesh cage in combination with cancellous bone allograft and demineralized bone matrix putty (Grafton) stabilized with an intramedullary nail. The titanium mesh cage was trimmed to 9 cm (diameter 2.2 cm) to fit the defect size and packed with allograft prior to implantation (**A**). Posterolateral callus consolidation around the cage on X-ray (views: (**B1**): anterior–posterior; (**B2**): lateral) 12 months post-reconstruction. Standing patient (**C1**) and standing on the operated limb (**C2**) at one-year follow-up. Adapted from Ref. [22], reproduced with permission from John Wiley & Sons, Inc.

The first clinical application of cylindrical (spinal) titanium mesh cages (DePuy Motech, Warsaw, IN, USA) was described in 1999 for combination with cancellous bone allograft and demineralized bone matrix for the reconstruction of posttraumatic diaphyseal tibial defects in two patients [123]. The mesh cages were cut to a length circa one cm longer than the defect and reinforced at both ends with internal rings attached to the cage. The authors reported that both patients reconstructed with the spinal titanium mesh cage were able to begin unrestricted weight-bearing early and achieved complete functional recovery with restored limb alignment [123]. Moreover, bony consolidation of the defect was confirmed one year postoperatively by plain radiography and computed tomography (CT) imaging, which confirmed bone healing circumferentially within the mesh and at the interfaces between the host bone and the cage [123]. Thus, this new single-stage reconstruction method using cylindrical modified spine titanium mesh cages loaded with allograft in combination with internal or external fixation immediately restored bone continuity, alignment, and stability (Figure 4).

**Figure 5:**
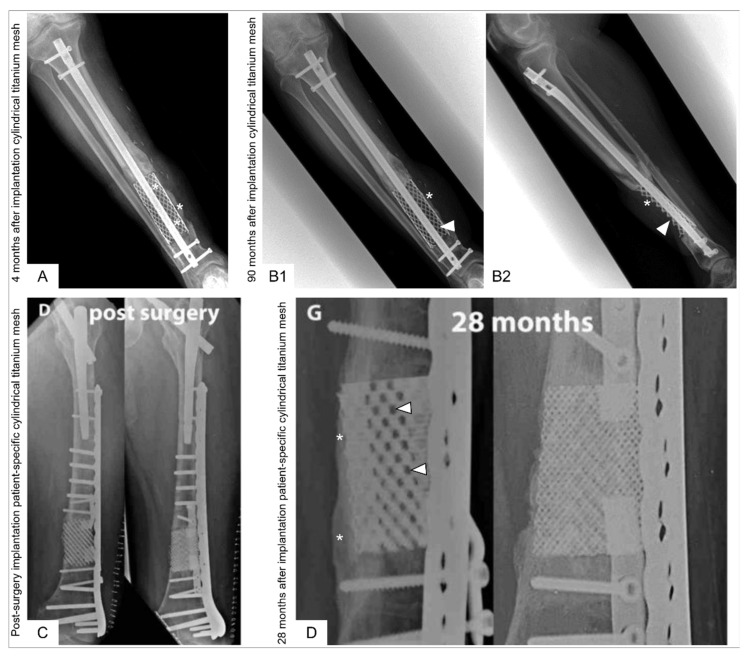
Exemplary challenges and failures in the use of cylindrical titanium mesh cage implants for bone defects. X-ray of a 29-year-old male with a segmental tibial defect and treatment for definitive fixation with an intramedullary nail and cylindrical titanium mesh cage (DePuy Synthes) (**A**). The asterisks in A1 highlight the graft material four months after grafting, which is packed into and on the outer surface of the cylindrical mesh implant. After 90 months, there is stagnation in the bony consolidation on the surface of the implant (asterisks) compared to plain radiography findings after four months and, in particular, no evidence of a bony structure within the cylindrical titanium mesh cage (triangles) ((**B1)**: anterior–posterior; (**B2**): lateral). Patient-specific titanium mesh implant (DePuy Synthes) packed with RIA system graft material implanted to treat a femoral defect (**C**). Twenty-eight months after implantation, a subtle but distinctive callus formed bridging the defect on the implant surface ((**D**), asterisks indicating bone bridge). However, no bony consolidation was observed in the inner part of the titanium mesh. The triangles in D indicate that the mesh structure is radiolucent, which would not be possible if ABG remodelling and thus successful regeneration of the graft material of the RIA system had occurred. (**A**,**B**): reproduced from Supplement of Ref. [126], with permission from the British Editorial Society of Bone & Joint Surgery; (**C**): reproduced from Supplement of Ref. [129], reprinted with permission from American Association for the Advancement of Science (AAAS).

Further reports followed on the use of modified spine titanium mesh cages with allograft [22,124,125,126] as well as the combination of cylindrical titanium mesh cages with ABG [126,127] or ABG supplemented with rhBMP-2 [128]. Following these case reports, major trauma centres worldwide began to apply cylindrical titanium mesh implants either as the previously described cylindrical (spinal) titanium mesh cage (DePuy-Synthes, Warsaw, Indiana) as reported in 17 patients by Attias et al. [126] or as a cylindrical 3D additive manufactured titanium mesh cages as reported in 19 patients by Pobloth et al. [129]. Attias et al. [126] implanted the non-patient-specific cylindrical titanium mesh cages (mean length 8.3 cm, range 2.6 to 13 cm) as an adjunct to either an intramedullary nail or plates and filled with allograft (13/17), Reamer-Irrigator-Aspirator (RIA) system graft material (2/17), and combination RIA system graft material/allograft (2/17). Device failure was observed in two patients (12%), and four patients (24%) had a residual limb-length discrepancy of a mean 1.5 cm (range 1 to 3 cm). Pobloth et al. [129] implanted patient-specific cylindrical 3D additive manufactured (selective laser sintering, SLS) titanium mesh cages (DePuy Synthes) augmented with either RIA system graft material or stuffed with a fibula transfer [129]. The authors report on two representative patients in detail while it was stated that “not all patient cases showed consistent bone defect bridging” [129]. Bony integration at the implant–bone interface was observed in the two detailed case descriptions, but no evidence of bone formation throughout the titanium mesh implant 28 months (case 1) and 8 months (case 2) after implantation [129] was observed. Challenges associated with cylindrical titanium mesh cages are depicted in Figure 5.

### 3.3. Importance of Graft Material Compartmentalization

The cylindrical mesh implants, which lack an interconnecting pore structure, therefore exhibit inadequate graft retention, resulting in excessively rapid graft remodelling, and it can thus be noted that these implants, by virtue of their morphological implant architecture, contribute to an osteoimmunological microenvironment most similar to that of 2.5D implants. The development of an alternative implant design that allows optimized and standardized packing as well as support of the bone graft was achieved with fully interconnected (3D) scaffold pore architectures (for definition of 2.5D and 3D implants, see Table 1—Definition 2). Notably, this has already been indicated in earlier large animal studies showing more effective reconstitution of the graft in a cylindrical polylactide double mesh despite its smaller volume compared to a single mesh [22,120] (Figure 3). This suggests that accessibility of the graft to nutrients from the adjacent soft tissue is important and that favourable biomechanical conditions exist for a porous implant-retaining bone graft rather than filling the entire defect, as is the case with a single mesh [22]. The entrapped graft is subjected to a direct load transmitted from the cortical bone and surrounding soft tissue between the dual mesh, whereas in the single mesh, the centre is not loaded [22]. This biomechanical difference in graft loading may contribute to more favourable graft reconstitution in the implants’ porous architecture, eventually leading to the concept of loading bone graft materials in pores of biomaterial implants. Thereby, the implant architecture provides a mechanical barrier and compartments for the graft material, protecting the bone graft from resorption and overly fibrous ingrowth. Therefore, 3D-printed scaffolds, equipped with ABG in their large pores, and fabricated from both non-biodegradable [10,129] and biodegradable [27] biomaterials with fully interconnected pore architecture network, may result in improved functional bone regeneration. The definition of the required structural features for the structures we now call ‘scaffolds’ was probably first established by Yannas and Burke [130,131]. In a series of studies and publications [130,131], Yannas and Burke described a highly porous analogue of the extracellular matrix based on type I collagen with specific structural features, which they called the “dermis regeneration template” (DRT) in their work.

## 4. Bone Defect Regeneration in the Era of Advanced 3D-Printing Technology Platforms

**Figure 6:**
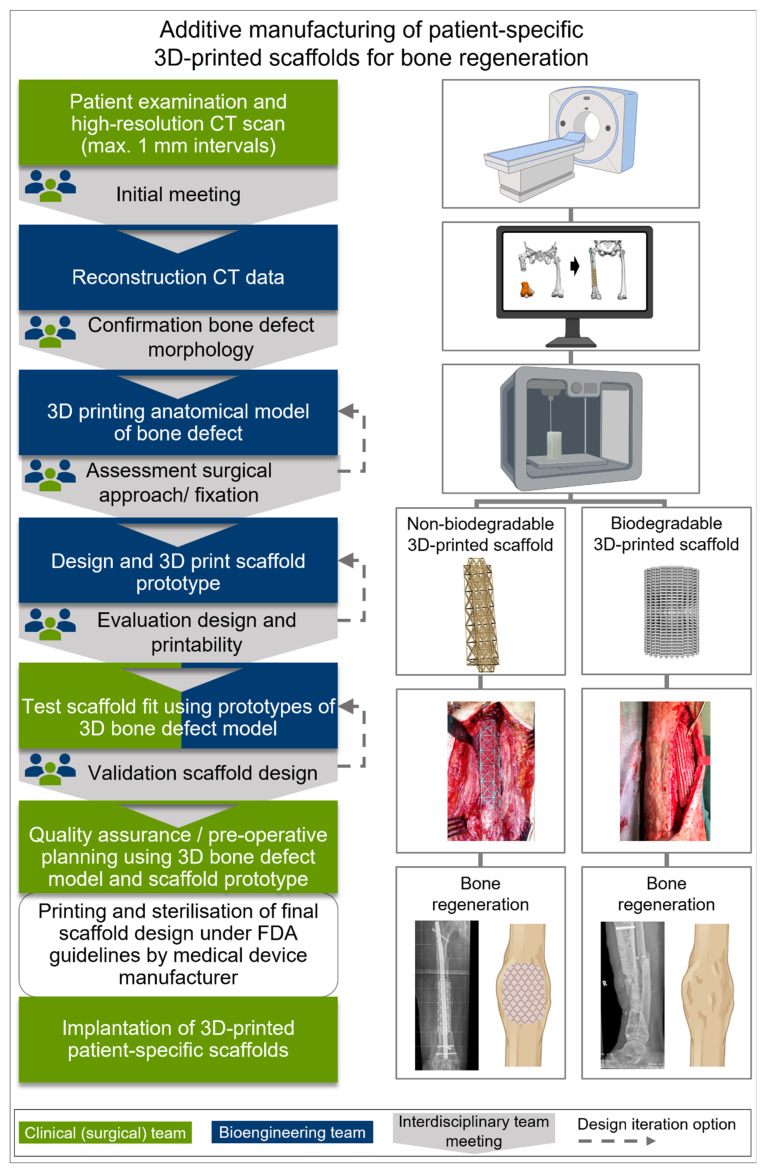
Workflow for image-based design, 3D printing, implant deployment and key bone regeneration differences depending on the biomaterial. Patient-specific 3D-printed titanium scaffolds are manufactured using electron beam melting (EBM) technology with medical-grade Ti-6Al-4V powder. The medical-grade PCL–TCP scaffolds are fabricated by fused deposition modelling (FDM). Adapted from Refs. [10,137,148,149]. The biodegradable 3D-printed scaffold is reprinted from Ref. [150], with permission from Elsevier. Partially created with BioRender.com.

In orthopaedic surgery, the convergence (merging of multiple parts such as technologies and disciplines into a unified whole [132,133]) of these advanced TE&RM sciences with the current high level of interest in developing applications for 3D modelling and 3D printing is leading an increasingly large number of surgeons to incorporate 3D modelling and virtual procedures into their routine clinical practice [134,135,136]. In the past, complex bone defects have required the use of improvised and modified implants that were initially manufactured for different anatomical sites and indications and, therefore, have a corresponding, specific complication profile when used for a purpose other than originally intended (Figure S1). Contemporary state-of-the-art printing technology allows for customized (patient-specific) non-biodegradable 3D-printed scaffolds as well as biodegradable 3D-printed scaffolds resulting in SGBR, both using 3D-printable biomaterials manufactured according to additive manufacturing (AM) principles [10,137] (Figure 6). This personalized therapy of long bone defects with 3D-printed medical devices has the advantage that the implants can be created exactly according to the defect morphology, and thus, firstly, mechanobiological optimized scaffold designs can be investigated; and, secondly, no further bone resection is necessary in the process of surgical implantation [10,23,129,137,138,139,140]. The fully interconnected large pores of the 3D-printed scaffolds provide an implant morphology with suitable microenvironment for guidance of the tissue regeneration [141] that facilitates diffusion of oxygen and nutrients, thereby supporting cell adhesion and proliferation, which are ultimately required for functional bone regeneration [142,143,144]. Therefore, the architectural hierarchies of non-biodegradable as well as biodegradable highly porous 3D-printed implants are transformed into biofunctionalities, such as osteo-immunomodulation [145] and osteointegration [146,147], which have been shown to facilitate the regeneration of long bone defects [137,140].

### 4.1. Additive Manufacturing and Surgical Utilization of Implants for SGBR

AM technology can be used for rapid prototyping and customization of non-biodegradable [10,140] and biodegradable [23,137] scaffolds. Detailed information on virtual surgical planning and interdisciplinary design development for 3D implants for the treatment of segmental bone defects is presented elsewhere [10,137,139]. Briefly, digital models with precisely defined lattice information are necessary to control the subsequent AM processes [149]. The concept of image-based design uses non-invasive imaging data, particularly CT images and numerical modelling for the design of custom 3D-printed implants [151,152]. To achieve high 3D implant design accuracy, CT scans of the patient’s long bone defect should be taken in increments of no more than 1 mm [10,137,140]. This approach integrates medical imaging, image processing, and 3D surface modelling to create patient-specific 3D implant designs with customizable internal architectures and porosities [148,153,154]. In short, scanned images of defective extremities are used to create virtual 3D models, which are then segmented, and after further processing, a digitized 3D surface model is obtained to design the internal pores of the 3D implant. In addition, the topological detail of the 3D implant is defined by the voxel density distribution of the CT scan. The layer-by-layer manufacture of the 3D model is then coded into a tool path file (e.g., G-code) to instruct the fabrication of the desired 3D implant, which is eventually implanted into the patient to regenerate defective bone. In addition, anatomical models of the bone defect can be 3D-printed by creating tangible 3D replicas of individualized patient anatomy and the prototypes of the implants used for thorough preoperative surgical planning, including surgical access, implantability, as well as fixation with orthopaedic implants [137,155]. Therefore, patient-specific 3D-printed scaffolds preserve the length and space of the defect, and, when additionally adding solid parts, can be attached to the proximal and distal ends of the host bone [137].

The design and fabrication of the patient-specific 3D-printed scaffolds are described for both non-biodegradable [10,140,156] and biodegradable [23,137,157] 3D implants as an interdisciplinary process that involves orthopaedic and design engineers (design team), the clinical (surgical) team, and the implant manufacturer, incorporating the planned surgical approach, screw trajectories, and fixation devices into the design. The workflow including virtual surgery planning for image-based design, manufacturing, and deployment of 3D-printed implants and anatomical models is shown in Figure 6.

The chosen strategy for bone defect treatment should be cost-effective, allow early functional rehabilitation, provide acceptable medium- as well as long-term outcomes, have an acceptable risk–benefit ratio, and respect local expertise and resources [6]. Respecting these premisses, biomaterials that have been used to fabricate porous patient-specific 3D-printed scaffolds with fully interconnected strut architecture for bone regeneration are medical-grade titanium-based biomaterials as well as composite scaffolds made of medical-grade polycaprolactone (mPCL) in combination with the calcium phosphate-based ceramic filler of tricalcium phosphate (TCP). As with conventional treatment methods with the therapeutic goal of bone regeneration, (potential) local and systemic infections must be treated and eradicated prior to implantation of 3D-printed scaffolds, and debridement of the sclerotic bone to healthy bone (e.g., indicated by bleeding cortical margins) must be achieved, always keeping in mind the key factor of adequate soft tissue coverage, which may require a multidisciplinary approach [43,137,158,159] (Figure 2). The bone graft or graft substitute is tightly packed into the 3D-printed scaffold pores, with the graft material extruding from the fenestrations once it is sufficiently packed [5,23,137]. The grafted 3D-printed implant is firmly compressed at the sites of contact with the host bone cortex and fixed in place with either a nail or a plate. The remaining graft material may be liberally placed along the periphery of the 3D-printed implant and at the implant–muscle interface, as well as at the junctions between the implant and host bone. Table 2 shows a selection of clinically relevant studies using 3D-printed scaffolds with a fully interconnected strut architecture for long bone defect regeneration.

### 4.2. Patient-Specific 3D-Printed Titanium Scaffolds

Early case reports on the use of patient-specific 3D-printed titanium scaffolds mainly involved complex cases with distal tibia and foot pathologies, including smaller bone defects of the foot [160,161,162], ankle arthrodesis [162,163,164,165,166], or reconstruction of a large distal tibial defect [162,167,168]. Noteworthy, the 3D-printed titanium scaffolds have multiple United States and international patents and are FDA-approved as patient-specific custom devices on a “compassionate basis” [10].

Titanium’s modulus of elasticity (>100 GPa [169]) is far from comparable to that of natural bone (3–30 GPa for cortical bone; 0.02–2 GPa for trabecular bone [170]), potentially resulting in stress shielding and undesirable resorption of adjacent bone [171]. Additionally, the prospects of bioinert titanium are limited because it can hardly fulfil the criteria of osteointegration and anti-infection [172]. Several solutions have been evaluated, such as (i) pre-print 3D architecture optimization [173] and (ii) titanium alloy development such as in combination with vanadium, aluminium, iron, etc. (e.g., Ti-6Al-4V) [174,175]). In particular, powder-based 3D-printed titanium alloys, especially Ti-6Al-4V, have dominated research in the field of metallic 3D printing [176,177,178] and are often chosen as implant materials for load-bearing applications due to their low density, high strength, and preferable biocompatibility [179]. Historically, biocompatibility was defined as the ability of a biomaterial to elicit an appropriate host response when used in a specific application [180]. Yet, this definition requires a fresh scientific ‘Zeitgeist’ which is beyond the scope of this review. As studies published to date outline [10,140,156], the titanium scaffolds used for the segmental defects were 3D-printed from medical-grade Ti-6Al-4V powder using the electron beam melting technique [181]. Titanium-based alloys such as Ti-6Al-4V exhibit good biocompatibility, reliably high mechanical strength, and exceptional resistance to fatigue loading and biocorrosion [182].

**Figure 7:**
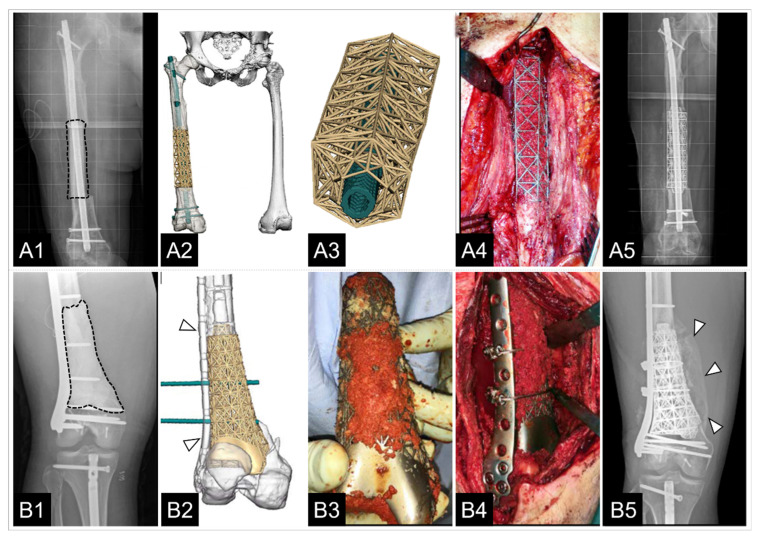
Two exemplary cases of femoral segmental defects reconstructed with 3D-printed titanium scaffolds. An infected diaphyseal femoral segmental defect (15.2 cm) was treated with the Masquelet technique. X-rays showing an antibiotic-loaded PMMA spacer (surrounding dashed line) fashioned to completely fill the defect, enveloping the bone at both the proximal and distal ends (**A1**). Three-dimensional-printed titanium scaffold implantation with 3D virtual procedure images showing the 3D implant, designed to allow stabilization by an intramedullary nail (**A2**). Please note that the 3D implant design incorporates an axial hole designed to fit the intramedullary nail for stabilization (**A3**). Intra-operative image during the second stage of the Masquelet technique, following insertion of the 3D-printed titanium implant with graft material packed into the large pores (**A4**). Anterior–posterior plain radiograph illustrating the final position of the implant, with a nail inserted through the 3D-printed scaffold locked proximally and distally (**A5**). Another distal femoral segmental defect (15.1 cm) was again treated with the Masquelet technique with anterior–posterior radiography following stabilization with a lateral plate and an antibiotic-loaded PMMA spacer (surrounding dashed line) (**B1**). The patient-specific 3D titanium scaffold design included trajectories for screw holes (green rods) that correspond to the existing plate (indicated with triangles) (**B2**). The large open pores of the 3D-printed titanium scaffold were manually packed with autologous and allogeneic cancellous bone graft material mixed with powdered vancomycin (**B3**). Intra-operative image during the second stage of Masquelet technique, showing additional graft material placed over the anterior and medial aspects of the implant following the completion of definitive fixation (**B4**). Anterior–posterior plain X-ray demonstrating early incorporation of the bone graft material, with a solid column of dense bridging bone visible medially (triangles) four months after implantation (**B5**). Figure adapted with permission from Ref. [10].

In 2019, the first preliminary results of a larger series of cases using patient-specific 3D-printed titanium scaffolds were published, highlighting their potential as another treatment option for complex segmental bone loss [140]. Moreover, in the majority of cases with 3D-printed titanium scaffolds used for segmental defects, it is stated that the implants provide sufficient strength and stability to allow early ambulation and protected weight-bearing [10,140,156]. The authors described achieving “very acceptable clinical outcomes” [140] and “progression to functional union” [156] in all cases (Figure 7). Furthermore, 3D-printed titanium scaffolds offer many mechanobiological advantages by facilitating bony integration at the bone–scaffold interface and throughout the scaffold [10,140,156]. Gamieldien et al. [156] attribute these sound clinical performances specifically to the modulus of elasticity of the 3D implants with octahedral configuration, which, on the one hand, represents a biomechanically advantageous construct with low density, but, on the other hand, also allows loading of the 3D-printed scaffold with graft material over the entire defect size [183,184].

**Table 2:** Selection of clinically relevant studies with additively manufactured patient-specific 3D-printed scaffolds with a fully interconnected pore network for the treatment of segmental long bone defects. RUST, radiographic union score for tibia.

Reference (Year)	Number of Patients (Mean Age and Range)	Anatomical Location	Pathology	Defect Size	Masquelet Technique	Implant for Fixation	Bone/Synthetic Graft Substitute	Perioperative Complications	Patient Outcome	Follow-Up
**Case studies with patient-specific 3D-printed medical-grade titanium (Ti-6Al-4V powder) scaffold using the ‘electron beam melting (EBM)’ printing technology**										
Tetsworth et al. [140] 2019	N = 5 (49.0 years; 26–73)	Femur	Post-traumatic defects	Mean length 14.0 cm (10.3–18.4 cm); mean volume 192.4 cc (114–292 cc)	Two-stage Masquelet technique	Intra-medullary nail or lateral locked plate	Anterior iliac crest bone graft/graft material harvested with RIA system/allograft cancellous chips	No deep infections, fractures, nerve injuries, loss of alignment, or non-unions identified during follow-up	All patients achieved union clinically and radiographically. At latest follow-up, all 5 were ambulating, fully weight-bearing, and pain-free, with 1 patient using a cane when ambulating distances.	21.8 months (range 12–33 months)
Gamieldien et al. [156]	N = 9 (36 years; 19–52)	Femur (n = 7); tibia (n = 2)	Chronic osteo-myelitis (n = 3); acute trauma with bone loss (n = 3); infected non-union (n = 2); aseptic bone defect non-union (n = 1)	Mean length 9.6 cm (3–20.5 cm)	8/9 patients two-stage Masquelet technique	Intra-medullary nail	Graft material harvested with RIA system (n = 8)/Posterior iliac crest bone graft (n = 1)	No peri- or post-operative complications occurred	All cases progressed to functional union at a mean of 3.1 months (range 2–4.6 months). RUST score [185]: 4/9 union at a mean of 4.9 months (range 2.6–7 months). 6/9 features of radiological union with callus enveloping both ends of truss at a mean of 4.9 months (range 2.6–7.4 months)	11.3 months (range 4.6–29 months)
Case report and case studies with patient-specific 3D-printed medical-grade PCL-TCP scaffolds using ‘fused deposition modelling (FDM)’ printing technology										
Kobbe et al. [23] 2020	N = 1 (29 years)	Femur	Post-traumatic defects	Circum-ferential bony defect, 6 cm at medial and 11 cm at lateral aspect of femur	No Masquelet technique	Intra-medullary nail	Graft material harvested with RIA system/rhBMP-2	None reported	Advanced bone fusion at scaffold–host bone interface and bone formation, both inside and outside the fully interconnected scaffold architecture.	12 months
Laubach et al. [137] 2022	N = 4 (23–42 years)	Femur (n = 2); tibia (n = 2)	Post-traumatic defects	Volume 29.89 cm^3^–165.72 cm^3^	Two-stage Masquelet technique	Plate or external fixator	Graft material harvested with RIA system/rhBMP-2/Cerament G	No peri-operative adverse events	In all cases, scaffolds matched the actual anatomical defect well; 3/4 cases showed evidence of bone ingrowth into the large honeycomb pores and fully interconnected scaffold architecture with indicated bony bridges 8–9 months after implant placement. In 1/4 cases, extensive bone regeneration and full loading capacity was achieved after 23 months.	8–23 months
Castrisos et al. [157] 2022	N = 2 (Case 1: 16 years; case 2: 27 years)	Tibia	Case 1: Ewing sarcoma; case 2: Osteo-myelitis	Volume case 1: 64.641 cm^2^; Volume case 2: 149.285 cm^2^ (length 36 cm)	No Masquelet technique	Case 1: Load bearing intra-medullary nail; Case 2: Load bearing plate and screws	Vascularized cortico-periosteal-cutaneous flap (CPCF) plus rhBMP-7. Case 1: ipsilateral medial femoral condyle; Case 2: 2x CPCF (1. Ipsilateral fibula; 2. Contralateral medial femoral condyle)	No intraoperative complications; Case 1: post-OP day 2: extensive blistering of the native skin distal to the CPCF skin paddle; Case 2: 19 months post-OP: Revision with IC ABG and 8 mL of rhBMP-7 for 6 mm defect at junction middle-distal third of the reconstruction	Case 1: good volumes of regenerated bone with osteosynthesis to native bone. Case 2: regenerated bone developed throughout the scaffold over 24 months.	Case 1: 24 months; Case 2: 48 months

The assessment of bone healing, for example in the context of the diagnosis of a non-union, as well as the general follow-up controls after bone defect treatment are typically based on clinical and radiological criteria. It appears that radiographic bone union in titanium scaffolds is difficult to define using the traditional standards of bridging callus formation over three or more cortices [156]. In addition, it also seems challenging to visualize bone formation within titanium scaffolds with plain radiographs as well as CT scans (Figure S2) [10,140,156]. Of note, although a CT scan might be helpful during follow-up to visualize radiographic union, its use is not regularly clinically indicated, especially in patients who achieved functional long bone union. In addition, the wear and corrosion of metallic implants can produce particles and toxic metal ions in the long term, which cause harmful chronic inflammation [149]. These concerns have led to the development of 3D-printed biodegradable scaffolds for tissue regeneration, which particularly benefit patients with long bone defects who have a high life expectancy and are subject to heavy physical stress, as these 3D implants degrade after implantation, and their degradation by-products are excreted by the organism to ultimately achieve bone regeneration [27].

### 4.3. Patient-Specific 3D-Printed mPCL-TCP Scaffolds

As an alternative to metallic implants, polymers consisting of covalently bonded long-chain repeating units in particular have been intensively studied over the last 50 years [149]. These macromolecules, in close association with recent advances in AM-printing technology, have several advantages that make them an important class of 3D scaffold biomaterials [186,187,188]: biocompatibility, light weight, functional structures, tuneable degradation, and high manufacturability [189,190,191]. Polycaprolactone (PCL) and PCL-based scaffolds are approved by the FDA and are accordingly used in the context of implants for tissue regeneration [192,193]. For example, a clinical study reported on a variety of craniotomies with burr holes and regeneration with an mPCL implant (Osteoplug and Osteoplug-C; Osteopore International Pte Ltd.) with a diameter of 12 mm and a thickness of 5 mm at a porosity of 70% including mPCL implants in 174 consecutive patients over a 10-year period with a mean follow-up of 248.1 ± 435.3 days [194]. Therefore, mPCL can be defined as a biocompatible synthetic polymer that degrades in the body over several years once new bone has formed to replace the scaffold [27]. For bone regeneration of a critical-sized segmental defect, based on a series of preclinical large animal studies [195,196,197,198], mPCL in combination with ceramic was identified as a particularly suitable biomaterial [199]. The incorporation of calcium phosphate-based ceramics, such as TCP, into the mPCL matrix results in a class of biomaterials (i.e., mPCL-TCP scaffolds) with improved mechanical properties, controllable degradation rates, and enhanced bioactivity. These materials have been extensively tested, and accordingly, marked osteoconductive and osteogenic capacity has been observed for these 3D-printed implants, including guiding template, facilitating cellular activities such as cell migration, recruitment, proliferation, and differentiation, all key components for successful bone regeneration [23,27,137,157,200,201,202].

**Figure 8:**
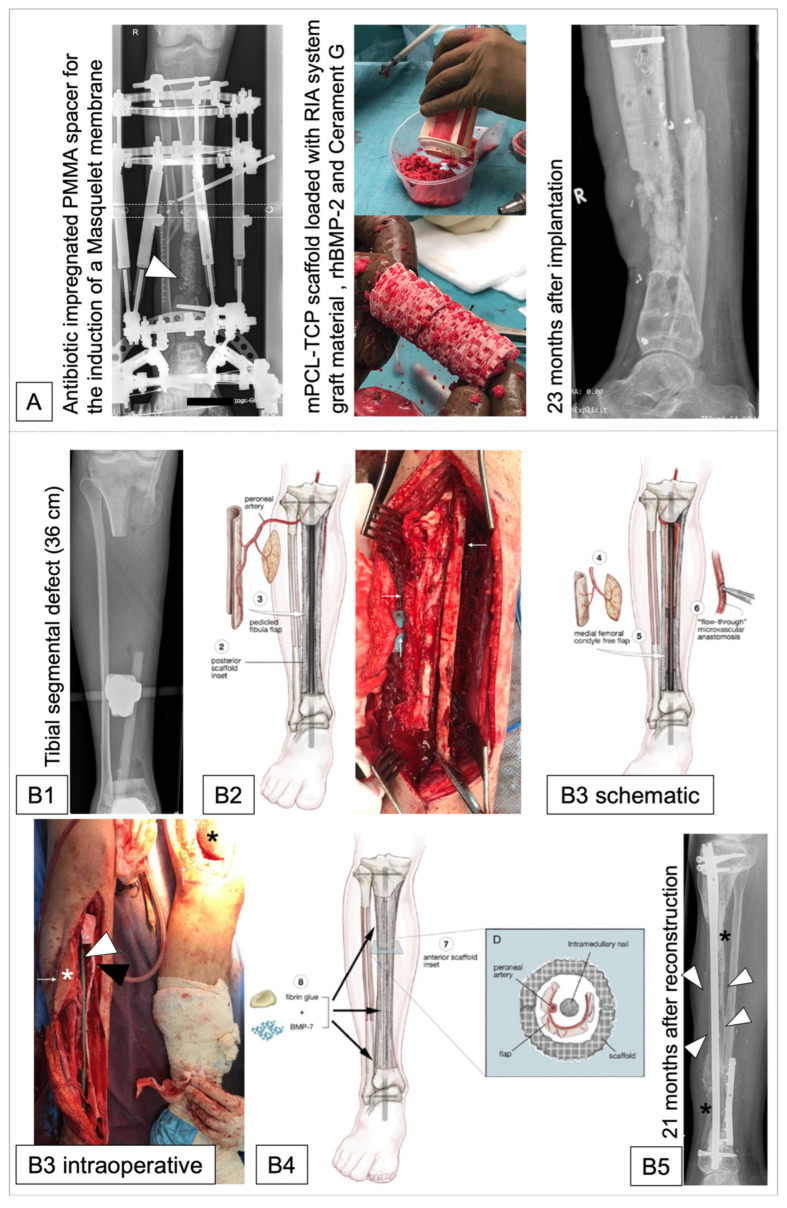
Two exemplary clinical cases treated with patient-specific 3D-printed mPCL-TCP scaffolds for segmental long bone defects. Treatment of a 10 cm tibial segment defect with an mPCL-TCP scaffold loaded with RIA system graft material, rhBMP-2, and Cerament G implanted into a Masquelet membrane ((**A**), white triangle indicates PMMA spacer in situ before its removal and scaffold implantation). Regenerative matching axial vascularization (RMAV) approach of mPCL-TCP scaffolds, impregnated with fibrin glue loaded with rhBMP-7, using corticoperiosteal–cutaneous flaps (CPCF) to achieve regeneration of a 36 cm near-total intercalary tibial defect secondary to osteomyelitis (**B**). Conventional radiograph of the patient’s lower extremity after resection of the clinical osteomyelitis that left a massive segmental defect of the tibia (**B1**). Schematic illustration of the surgical steps: a CPCF is harvested from the ipsilateral fibula (**B2**); the CPCF tunnelled to the anterior compartment and positioned inside the posterior component of the mPCL-TCP scaffold (white triangle indicates intramedullary nail; black triangle indicates posterior component of the mPCL-TCP scaffold; white asterisk indicates skin paddle of fibular CPCF). A second CPCF raised from the contralateral medial femoral condyle (black asterisk contralateral donor site), auto transplanted to the distal bony defect and anastomosed to the transposed peroneal vessels (**B3**). Centripetal neo-osteogenesis directed and supported by the mPCL-TCP scaffold (**B4**). At 21 months after reconstruction, integration of the mPCL-TCP scaffold at the proximal and distal ends of the defect (black asterisks) and bone formation within and outside the fully interconnected scaffold architecture (white triangles) were achieved (**B5**). (**A**): adapted from Ref. [137]. (**B2**–**B4**): reprinted from Ref. [157], with permission from Elsevier.

Notably, while other reviews focus on the progress of 3D-printed scaffolds for bone regeneration from a basic research perspective and particularly include information on topics such as bioprinting, bioactive scaffolds, and composite materials [149,203,204,205], this review aims in particular to address translational challenges which have been in large neglected in current literature. To date, the clinically used 3D-printed mPCL-TCP scaffolds have large pores, with >70–80% porosity, have a 0°/60°/120° strut lay-down pattern manufactured by Osteopore International Pty Ltd. (Singapore), and are gamma-sterilized before implantation [23,137,157]. After extensive in vitro and in vivo testing focusing on mechanical properties, degradation, geometry, surface and manufacturing optimization [199,206,207,208,209,210], and application of the implants in large animal trials on large to extra-large tibial segment defects in sheep [196,201,206,209,211], the first published case of grafted patient-specific porous 3D-printed mPCL-TCP, in the coined concept of SGBR for the treatment of a large femoral bone defect, dates to 2020 [23]. More recently, in a case series, patient-specific 3D-printed mPCL-TCP scaffolds were successfully used in combination with the Masquelet technique in patients with post-traumatic long bone defects [137]. Within a mean follow-up time of 8–23 months, functional bone repair was achieved in all cases with bone formation throughout the large honeycomb pores and fully interconnected scaffold architecture [137]. Notably, in a 10 cm tibial defect, complete bone remodelling with full weight-bearing capacity and removal of internal fixation implant (plates) was achieved after 23 months [137] (Figure 8A).

Recently, the SGBR principles were extended to the treatment of massive bone defects based on the concept of regenerative matching axial vascularisation (RMAV) using vascularized corticoperiosteal–cutaneous flap (CPCF) [132,200,212]. This concept may be described as a modified Capanna technique [213] in which a pedicled CPCF is transferred, inverted, and laid inside the 3D-printed scaffold. This technique has now been applied to the tibia in two patients with defects of 149 cm^3^ and 65 cm^3^ [157,212]. At 36 cm, described in Case 2 in Table 1 by Castrisos et al. [157], this is the longest segment of load-bearing bone ever successfully reconstructed, with the regenerated bone radiologically visible as early as nine months after repair and bone formation developing throughout the scaffold over 24 months (Figure 8B) leading to complete independent weight bearing within 24 months [212]. Therefore, the authors concluded that this technique may facilitate the regeneration of bone defects previously considered unreconstructable [157,212]. Although this technique offers a closed reconstructive system, it relies heavily on close interdisciplinary collaboration, the availability of microsurgical expertise, and careful postoperative follow-up [157,214,215,216]. However, these would be considered essential features of any quaternary referral limb reconstruction service worldwide. Whether or not the reduction in morbidity when compared with the Capanna technique and the potential health economic benefits of this style of reconstruction justifies the additional resources required remains to be seen. The place of RMAV in the reconstructive algorithm for large bone defects can be considered similarly.

## 5. Synergistic Effect of 3D-Printed Scaffolds with the Masquelet Technique and Bone Transport to Facilitate Bone Regeneration

Particularly in infected or potentially infected long bone defects, the application of the Masquelet technique with antibiotic spacer implantation after excision of any necrotic or infected bone will remain a remarkable feature and very valuable in the treatment [66]. However, case series studies of the treatment of tibial non-union defects applying the Masquelet technique have shown variable success rates and regularly required multiple additional surgeries [217,218,219,220]. Moreover, a meta-analytic estimate of the union rate independent of defect size using Masquelet technique in the tibia was found to be 84% (95% CI, 79–88%) [221]. Further, a direct comparison between the Masquelet technique and bone transport was performed by Rohilla et al. [222] in a recent prospective randomized study in which tibial infected non-union gaps of less than 6 cm were treated in both groups with an external fixation frame (either monolateral or circular). The union rate was significantly higher in the bone transport group than in the Masquelet technique group (92% vs. 50%), while the functional outcomes in both groups were comparable [222]. This is consistent with the observations of Mühlhäusser et al. [223], who found an increased risk of treatment failure of the Masquelet technique in bone defects with a volume greater than 80 cc.

It was Gugala and Gogolewski [120] who observed in a sheep study 25 years ago that bone graft firmly contained in two perforated (tubular) membranes resulted in enhanced radiographic and histologic bone regeneration compared to (even greater amounts of) ABG contained in the space of a single perforated (tubular) membrane (Figure 3). Therefore, the predominant role of the bone graft in bone regeneration may be facilitated by incorporation into highly porous implants for improved nutrition provided by the surrounding soft tissue and medullary canal. O’Malley and Kates [128] were the first to describe the hybrid technique, a single case in which a titanium mesh spinal cage (Stryker, Michigan, USA) was used in a two-stage reconstruction of Masquelet technique along with rhBMP-2 and graft material harvested with the RIA system. Following this case report, Tetsworth et al. [140] published an observational cohort study using a patient-specific 3D-printed titanium scaffold in conjunction with the Masquelet technique to reconstruct massive post-traumatic segmental femoral defects. According to Tetsworth et al. [140], the Masquelet technique’s staged approach was vital and played a critical role in the successful and stable osseous integration of the 3D-printed titanium scaffolds for three reasons: (1) it allowed for complete soft tissue recovery; (2) it provided the time necessary to complete the required additional processes for the design, fabrication, sterilization, and delivery of the custom 3D implants [10]; (3) the pseudo-membrane of the Masquelet technique allowed for a favourable environment with high osteogenic capacity.

Indeed, in vivo studies have shown that Masquelet membrane contains growth factors such as vascular endothelial growth factor, transforming growth factor-beta 1, and osteoinductive factor BMP-2 [224,225,226], as well as expressing a collagen-rich matrix, and could therefore enhance bone regeneration by facilitating the differentiation and proliferation of mesenchymal stem cells [227] toward the osteoblastic lineage [225]. Based on these findings, Laubach et al. [137] also reported on successful synergistic effects of improved graft material retention and containment resulting in increased quality of the regenerated bone when using large pore 3D-printed mPCL-TCP scaffolds in combination with the Masquelet technique, successful osseous implant integration, and adequate function of the limbs in follow-up. Therefore, for ABG-loaded 3D-printed titanium scaffolds [128,140,156] and mPCL-TCP scaffolds [137], placement within the pseudo-membrane during the second stage of the Masquelet technique proved feasible and was associated with good bone regeneration, which may further contribute to the quest with which graft components the pseudo-membrane should be filled [5]. Therefore, it can be concluded that the joint use of Masquelet membrane with 3D-printed scaffolds creates synergisms, and combined treatment contributes to the therapeutic success.

The Ilizarov method (bone transport) with either a circular or uniaxial external fixator has the advantage of not only addressing the problem of the osseous defect but also any malalignment, shortening, soft-tissue loss or joint contractures to restore long bone defects of the lower limbs (intramembranous ossification at distraction site [228,229]) with simultaneous eradication of longstanding bone infections [230]. Despite being associated with a multitude of complications [231], bone transport will remain an invaluable treatment option [232]. Bone transport involves corticotomy of the affected long tubular bone away from the injury zone and gradual transport of the bone segment to a docking site [66], achieving regeneration of the living bone with the same strength and width as native bone [232,233]. In bone transport, the use of additional bone grafts is common in cases of delayed union at the docking site or delayed maturation of regenerated bone (Figure S3) [230], and it is conceivable that 3D-printed scaffolds loaded with graft material may represent a complementary option to facilitate the docking process. Therefore, clinical studies may clarify whether 3D-printed scaffolds can facilitate union at the docking site, which is known to be a limited source of recruitable mesenchymal stem cells and particularly in long bone transports (>8 cm) with resultant dysvascularity as well as the informally coined “coat sleeve” phenomena [5] and may therefore benefit from 3D-printed scaffold’s additional osteoconductive and osteogenic support.

## 6. Patient-Specific 3D-Printed Scaffolds to Regenerate Long Bone Defects: Convergence and Clinical Translation

Combined with personal anatomical data and computer-aided topology design, recent 3D printing technologies enable virtually limitless design and on-demand manufacturing of complex, defect-shaped porous implants for bone regeneration [3,153,234]. In addition, 3D-printed scaffolds used for bone regeneration are designed in a virtual surgical environment that can provide additional patient-specific options for long bone realignment and length correction [10,139]. Thus, the unparalleled features of automation, speed, reproducibility, small-batch flexibility, and (potentially) low cost make recent developments in 3D printing for orthopaedic applications highly appealing and enable successful translation from bench to real-world applications [3,69,235,236]. Therefore, the convergence of AM and bone tissue engineering (BTE) has ushered in a new era of bone healthcare [236] in which patient-specific, anatomically matched 3D-printed implants are used in concepts such as SGBR for customized regeneration of defective or dysfunctional skeletal-related tissue. However, despite promising and widely published preclinical results worldwide, only a few approaches have been translated into routine clinical use, and this gap is referred to as the “valley of death” in the field of tissue engineering [237]. Furthermore, it is imperative to remember that publications are important dissemination tools as they provide critical reports of progress to stakeholders, but they are not an end product, as is often seen by researchers and scientists. Thus, there is a discrepancy between a large body of published research papers but limited translation of findings and (implant) developments into routine clinical practice. Henceforth, we aim to address this conundrum.

### 6.1. Torrential Stream following the “Valley of Death”

The gap (“valley of death”) between research and commercialization has been identified as such, as many companies “die” between technology development and actual commercialization due to a lack of funding. Remarkedly, relevant business challenges to translation described about 15 years ago in the “valley of death” [238] could be successfully addressed in the meantime: (1) regulatory approval in many countries could be negotiated, and patient-oriented solutions could be found; and (2) external funding with the financing of (confirmatory) large animal studies [239,240] as well as clinical studies [241] can be increasingly tracked. However, there is still a significant gap between the innumerable preclinical studies and the clinically applied scaffolds for the treatment of bone defects. In fact, even if the gauntlet of research, development, and regulatory approval is successfully run, this is no guarantee that a product will be clinically successful or even make it into clinical use, as influencing factors such as the decision-making processes of surgeons and (biomaterial) scientists need to be understood in detail. Therefore, understanding the decision-making processes of surgeons and (biomaterial) scientists regarding current options and future possibilities for treating bone defects is critical to (cost-)effective healthcare, keeping in mind that the total cost of bone reconstruction worldwide is expected to reach USD 17 billion [242], and global market for bone grafts and substitutes will grow at a compound annual growth rate of 5.8% from 2021 to 2028, reaching a value of USD 4.3 billion by 2028 [243].

Fundamentally, academia (scientist), clinics (surgeons), and businesses (industry) operate within very different environments or model systems. In academia, there is (unfortunately) a need to publish results quickly, and emphasis is placed on the ability to report on results or a technique first; there is often little, if any, reward for a researcher who emphasizes the research on reproducibility or the in-depth characterization of the technique/procedure or verifies and extends the original results, even if the modified procedure represents a significant leap from the original research [237]. In business and clinical practice, the reward is the end result of a product that sustainably improves patient care, not necessarily the first company on the market with an innovative product. In fact, the companies and clinicians that succeed are those that perfect the product, growing and consolidating the market, and not those who bring their products to market at the earliest possible time [244]. The obvious advantages of the concept of 3D-printed implants in the treatment of long bone defects include relatively simple surgical placement, immediate (one-step) restoration of bone continuity, and, in combination with internal or external fixation, stability that allows early resumption of functional activity of the affected limb. However, it is also evident that an interdisciplinary consideration and discussion of how novel treatment options such as 3D-printed scaffolds for the regeneration of segmental bone defects are perceived could enrich the research framework and inform those working in academia as well as in the field of biomaterials of challenges and opportunities [149,234].

### 6.2. Three-Dimensional-Printed Bone Regeneration Scaffolds—Quo Vadis? Consensus via Stakeholder Workshop

Currently, only one prospective clinical trial of a small study entitled “A Case Series: TRUMATCH Graft Cage for Segmental Long Bone Defects” is registered at clinicaltrials.gov (ClinicalTrials.gov Identifier: NCT05668182), testing a new, biodegradable 3D-printed graft material cage (DePuy Synthes’ TRUMATCH^TM^ Graft Cage (FDA 510 (k), K180821), Figure S4), and aims to enrol five participants. Thus, no sufficiently powered clinical trials on 3D-printed scaffolds for the treatment of long bone defects have yet been registered at clinicaltrials.gov, whereas 16,196 articles have been published in the last 30 years, with an annual increasing trend (Figure S5). However, a recent phase IIa clinical trial registered with the Australian New Zealand Clinical Trials Registry (ANZCTR) using the RMAV approach [conducted in Brisbane, Australia, at a major trauma centre (ANZCTR No. 12620001007921)] [245], provides hope for future results of larger clinical trials of 3D-printed scaffolds for long bone defects. Nonetheless, as described earlier by Hollister and Murphy [238], to routinely establish standardized large-scale multicentre (pre)clinical research efforts, we must first define the goal of BTE and the role that biomaterial implants play in achieving this aim—that is, we must find viable and both clinical and economical approaches to bone regeneration in orthopaedic surgery for long bone defects. In particular, there is limited guidance on the specific surgical indications, the current medico-legal status and reimbursement, and the (large-scale) clinical trials that should be performed [246,247].

Therefore, with new opportunities such as 3D-printed scaffolds for bone defect regeneration, it is equally important to achieve consensus among the leading stakeholders in this field to eventually obtain high-quality findings that will lead to guidelines applicable to all treatment centres worldwide [23,137]. The need for international consensus on surgical care is not new because, as Atul Gawande reported more than 10 years ago, researchers began documenting significant rates of fatal errors in surgical care, wide disparities in outcomes between institutions, and wide disparities in access to care both within the United States and between countries as early as the 1970s [248]. The treatment of bone defects, with their highly complex and challenging cases (the ‘holy grail’ of orthopaedics [96,249]), is certainly no different than the findings by Gawande published in the New England Journal of Medicine in 2012 [248].

Translationally meritorious convergence, which can be achieved, for example, by coordinated efforts from multiple parties conducting stakeholder workshops, must aim to narrow and close the gaps between scientific research and practical application and ultimately introduce technical innovations that change clinical routines and/or underlie new clinical therapy concepts [149,250,251]. Moreover, to break the prevailing, pervasive incentive system for scientific recognition, promotion, and success in academic research based on impact-agnostic numerical compilations and assessments of scientific output, to finally eliminate the perverse incentives of for-profit external forces [252,253,254,255,256], and to refocus biomedical research on the more altruistic and impactful goals originally envisioned beyond publications will require the concerted will and commitment of multiple stakeholders [254,257,258]. A stakeholder workshop may bring together clinicians (surgeons), biomaterial scientists, biomedical engineers, legal/regulatory professionals, experts for patient and stakeholder engagement, members of patient advisory boards, health economists, meta-researchers (who conduct research on research), artificial intelligence experts, and biomaterial industry members. These leaders in their respective fields would come together in a consensus meeting to discuss translational challenges by contributing their perspectives and potential solutions, without the constraint of their own (institutional) agendas, as this meeting is beyond their daily routines and (institutional) liabilities [259,260]. Within such a consensus meeting, the goal is to identify ways in which 3D-printed scaffolds can be used to achieve the key clinical and health economic means to the success of long bone regeneration therapy, as previously also identified by others [238]: (1) improved patient outcomes, (2) reduced morbidity or complications, and (3) reduced overall health care costs. To achieve this, a stakeholder workshop, including guided small group discussions, provides the opportunity to work towards a consensus on key parameters that urgently need to be addressed: optimization and synergistic potential in the areas of scaffold design, (pre)clinical studies, and regulatory process and reimbursement strategies (Table 3).

## 7. Conclusions

Although various long bone defect treatment methods are advocated, all current techniques are associated with major obstacles that include inadequate limb function, lack of patient compliance, serial surgical procedures, and/or the need for specialized equipment and skills [124]. It should be noted that there is no “one treatment fits all” solution in this difficult, challenging patient population, and that the patient’s profile, expectations, clinical history, and conditions of the local bone defect environment require an individualized treatment approach [269]. Notably, common treatment methods are also being refined, such as a modification of the classic distraction osteogenesis technique with a novel method of bone transport via an intramedullary nail using an intramedullary cable transport system [270,271], novel plate-assisted bone segment transport procedure [272,273,274], or the combination of bone transport with the Masquelet technique [275,276].

Thus, the indication and applicability of 3D-printed scaffolds for the treatment of long bone defects are still debated, and no consensus has been reached yet [277]. All adjuncts for the treatment of bone defects have their indications and limitations, and as such, 3D-printed scaffolds to regenerate bone defects can be a useful adjunct to the surgeon’s armamentarium. In our opinion, a stakeholder workshop is urgently needed to target prospective investigations of patient-specific 3D-printed scaffolds for the treatment of long bone defects in which surgeons, biomaterial scientists, biomedical engineers, legal/regulatory professionals, experts for patient and stakeholder engagement, members of patient advisory boards, health economists, meta-researchers, artificial intelligence experts, and biomaterial industry members may participate to discuss specific surgical indications, (pre)clinical trials that should be performed, the current medico-legal status, and reimbursement solutions. Especially due to the relative rarity of these pathologies, it is necessary to purposefully investigate 3D-printed scaffolds for long bone defect regeneration in large-scale, multicentre studies involving leading international orthopaedic centres for complex bone defects.

## Figures and Tables

**Table 1:** Definitions to distinguish the basic characteristics of ‘fracture repair’ and ‘segmental bone defect regeneration’ (Definition 1) and to distinguish ‘2.5D implant’ and ‘3D implant’ (Definition 2).

	Bone Fracture Repair	Segmental Bone Defect regeneration
Definition 1	- process by which bone tissue is repaired after a fracture or injury [106] - formation of a fibrous or cartilaginous callus that stabilizes the fracture site and facilitates bone repair - over time, the callus is replaced by new bone tissue as the fracture heals	- generation of new bone tissue in response to severe bone loss - process by which new bone tissue is formed without the formation of a callus [107] - this process can occur naturally in response to injury or disease, or it can be facilitated using various techniques, such as bone grafts and/or 3D-printed scaffolds
	**2.5D Implant**	**3D Implant**
Definition 2	- a 3D structure that has a depth significantly smaller than its length and width, giving the overall appearance of a thin, planar structure - the surface often contains some form of curvature or depth patterning [108,109] that differentiates it from a purely 2D substrate - does not provide sufficient space for cells to grow and differentiate into 3D neotissue [110,111,112] - biological factor diffusion is fast and passive [108] - microenvironment for seeded cells is a combination of static and dynamic [108] - can only be analysed only in two axes - fabricated using conventional replica moulding and lithographic techniques, electrospinning or bioprinting, and can be designed with some control over the structure and mechanical properties [113] - high surface-to-volume ratio. Stiffness-to-weight and strength-to-weight ratios can only be varied over a miniscule range by manipulating the surface architecture - they have no significant 3D porous architecture	- a 3D structure that has length, width, and depth and can provide a sufficient space for cells to grow and differentiate into 3D neotissue [110,111,112] - biological factor diffusion is slow and active [108] - microenvironment for seeded cells is dynamic [108] - can be analysed in all three axes - fabricated using techniques such as additive manufacturing or bioprinting and can be designed with precise control over the structure, porosity, and mechanical properties - surface-to-volume ratio, stiffness-to-weight ratio, and strength-to-weight ratios can be modified easily by changing the infill architecture and density [114] - macro mechanical properties of 3D porous scaffolds can be tuned by varying the pore size, pore shape, and strut thickness [115]

**Table 3:** Selection of key parameters in scaffold-guided bone regeneration for the treatment of long bone defects requiring interdisciplinary consensus.

Challenge	Interdisciplinary (Stakeholder Workshop) Approach
Design of patient-specific 3D-printed scaffolds	The optimization of scaffold design using machine learning [183,261,262] and finite element analysis [139,234,247] methods to avoid complications such as implant failure has been deemed required (Figure S6), especially when using titanium-based scaffolds, but is not yet fully integrated into current workflows [234].
Preclinical and clinical large-scale multicentre studies	Preclinical studies to increase research reproducibility [260,263] and for clinical studies in general in the field of large bone defect treatment using 3D-printed scaffolds can only be performed in purposefully planned and well-powered multicentre large-scale collaborative research projects to have the possibility to correct confounding factors related to host and soft tissue while investigating the respective implant for different defect sizes or for different surgical indications [157,264,265]. Furthermore, as bone defects are rare but very complex diseases with a dramatic socio-economic impact on the healthcare system, there are many open questions that may be better understood in the future through the use of artificial intelligence methods—from predictive models and cost analyses to personalised treatment strategies [266]; however, these methods first need to be validated in preclinical and clinical studies.
Regulatory process	Exemptions have been created for patient-specific 3D-printed scaffolds, allowing clinicians to quickly commission the manufacture of custom implants without having to undertake and comply with the complex and time-consuming regulatory process for each individual product; however, the need for strict reporting obligations and manufacturing transparency remains [267], which requires in-depth elaboration from medico-legal experts to reduce barriers and increase standardized use of 3D-printed scaffolds for bone regeneration [247].
Reimbursement strategies	The health economic impact of custom 3D-printed titanium and mPCL-TCP scaffolds for the treatment of large segmental bone defects remains to be defined. Only very limited data are available on costs for 3D-printed implants. An average cost of USD 2329 per 3D-printed titanium implant was reported by Gamieldien et al. [156]. Further, based our experience, approximate costs of mPCL-TCP scaffolds amount to USD 2700. It is important to point out that one of the main factors affecting the cost of 3D-printed scaffolds is the design time for each individual scaffold, and that this time is expected to reduce with increasing experience and new automated methods [149,261,262]. Therefore, in interdisciplinary (stakeholder workshop) meetings, it is a *conditio sine qua non* for the planning of future studies to include the variables of assessing the direct and indirect costs of different materials and treatment methods for 3D-printed scaffolds for long bone regeneration [268].

## Data Availability

Not applicable.

## Supplementary Materials

The following supporting information can be downloaded at: https://www.mdpi.com/article/10.3390/jfb14070341/s1, Figure S1: Cylindrical titanium meshes, originally developed as implants for spinal fusion, were used to treat a segmental defect in the distal femur; Figure S2: Computer tomography (CT) scan images taken nine months postoperatively after implantation of the 3D-printed titanium scaffold—graft construct; Figure S3: Plate-supported bone segment transport (antegrade) and docking site procedure in an exemplary patient; Figure S4: TruMatch™-Graft-Cage (Depuy Synthes, Umkirch, Germany) is a commercially available scaffold made of biodegradable polycaprolactone (PCL, 96%) and hydroxyapatite (HA, 4%) coated with calcium phosphate; Figure S5: Annual growth of studies on scaffolds for bone tissue engineering published in PubMed between 1996 and 2023; Figure S6: Depiction of a patient-specific Ti-6Al-4V-based implant after bone resection of a Ewing sarcoma with fracture at the proximal implant-bone interface one year after surgery due to a traumatic fall and retrospective finite element analysis (FEA) to investigate implant failure.
