# Supplementary material for: The Concept of Scaffold-Guided Bone Regeneration for the Treatment of Long Bone Defects: Current Clinical Application and Future Perspective
Source: J Funct Biomater. 2023 Jun 27;14(7):341. doi: 10.3390/jfb14070341 (PMC10381286; doi:10.3390/jfb14070341)
Supplement: Supplementary file 1 [file jfb-14-00341-s001.zip › jfb-2381635-supplementary.pdf]

Journal of
Functional
Biomaterials

Supplement S1

Figure S1. Cylindrical titanium meshes, originally developed as implants for spinal fusion, were used to treat a segmental defect in the distal femur. In the intraoperative image, it is evident that two cylindrical titanium meshes were required because the maximum length of one implant is insufficient to reconstruct a distal femoral defect (one white line per implant in A). Therefore, two stacked mesh cages were implanted with allograft (A). An anterior-posterior x-ray reveals that the titanium mesh cages are canted (white triangle), resulting in defect healing with new bone formation spanning the defect mainly along the medial side of the construct (black asterisks) (B). Three years after implantation, coronal (C) and axial CT images (D) confirm complete bone consolidation, but an implant design not dedicated to treatment of segmental bone defects resulted in suboptimal biomechanical stimulation, leading to unilateral bone consolidation (black asterisk in C and D). Adapted from Ref [1], reproduced with permission from John Wiley & Sons, Inc.

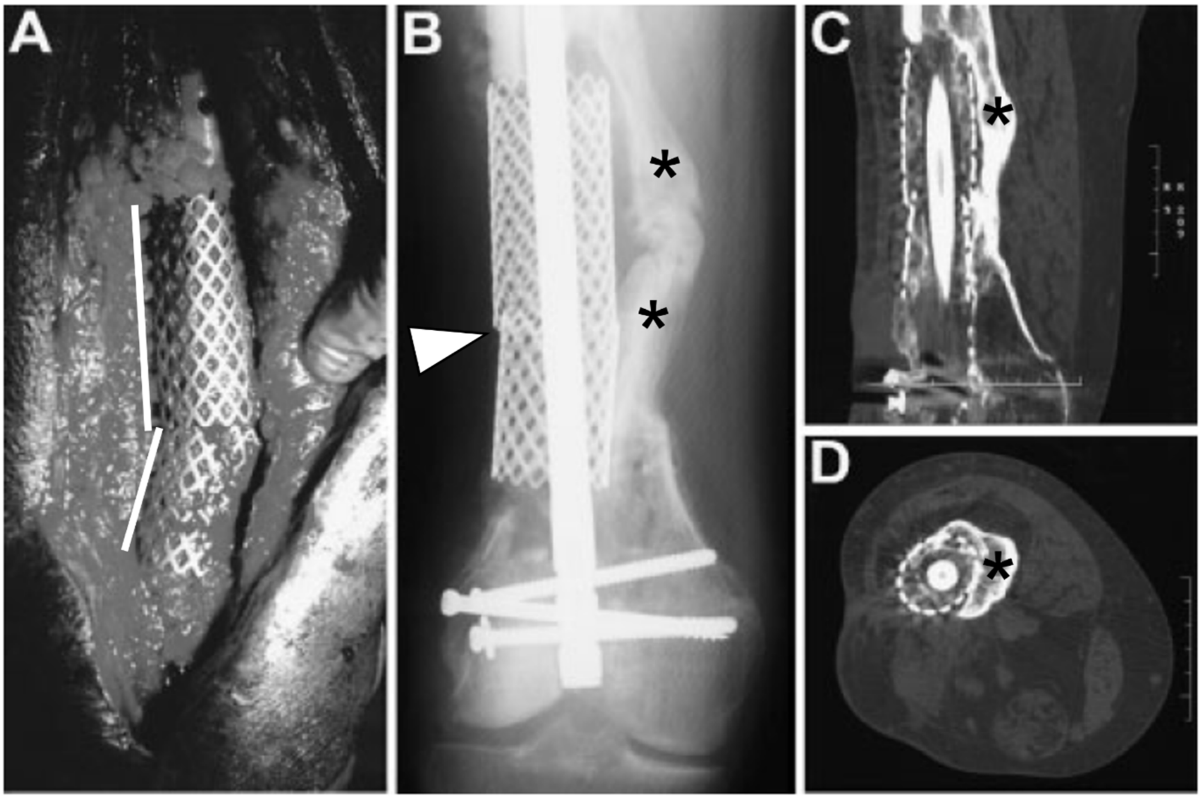

Figure S2. Computer tomography (CT) scan images taken nine months postoperatively after implantation of the 3D-printed titanium scaffold - graft construct. Please note that metal artifacts obscure many details related to bone formation throughout the 3D titanium implant. Reproduced with permission from Ref [2].

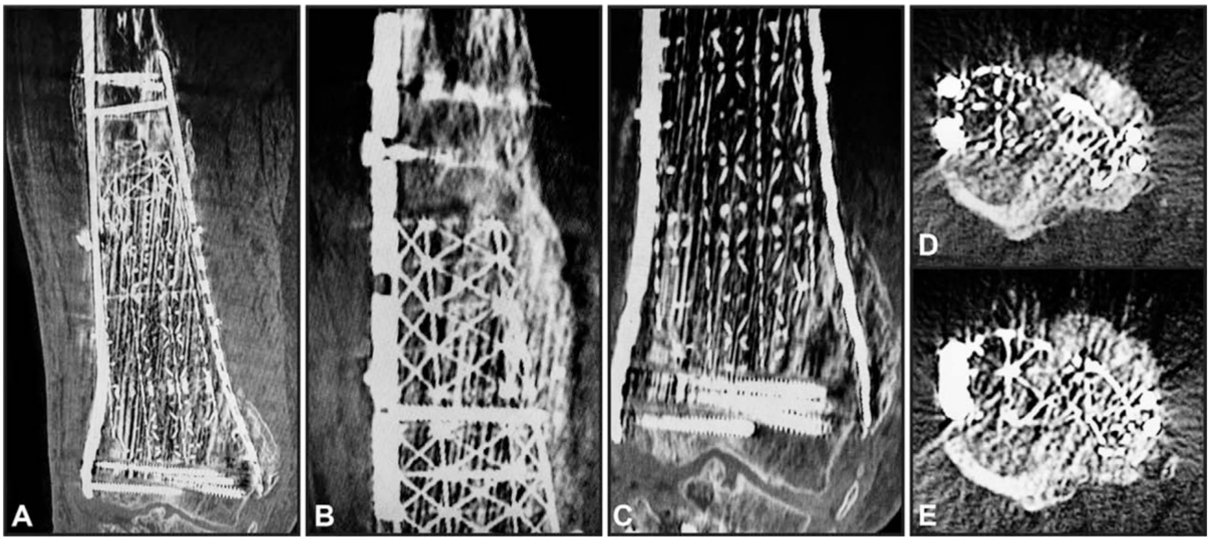

Figure S3. Plate-supported bone segment transport (antegrade) and docking site procedure in an exemplary patient. X-ray of the left femur after completed antegrade transport over an intramedullary nail (A), in which the surgical team indicated the addition of autologous cancellous bone graft at the docking site (red rectangle in A) and additive plate osteosynthesis (B) to facilitate bony consolidation of the docking site (white triangles in B). Adapted from Ref [3].

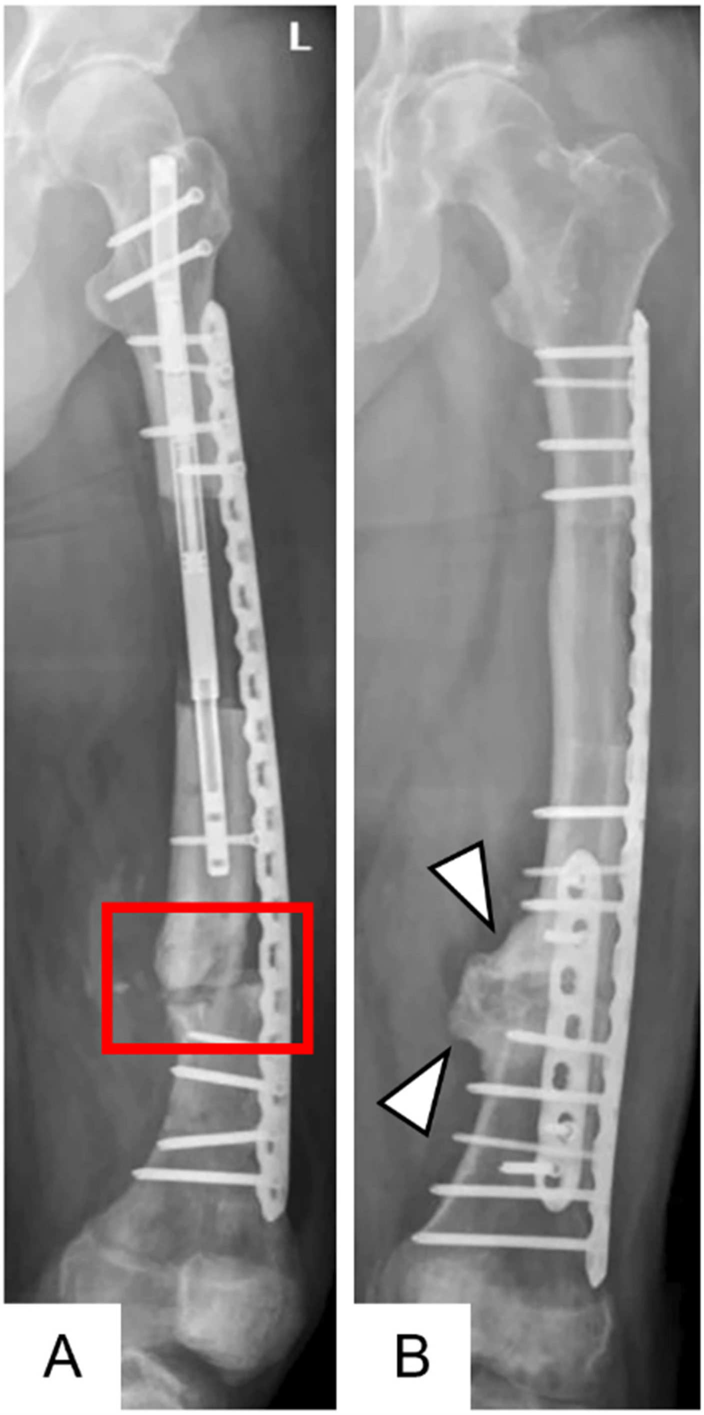

Figure S4. TruMatch™-Graft-Cage (Depuy Synthes, Umkirch, Germany) is a commercially available scaffold made of biodegradable polycaprolactone (PCL, 96%) and hydroxyapatite (HA, 4%) coated with calcium phosphate. View from the front (A), view from the cranial (B). Reprinted with permission from Ref [4].

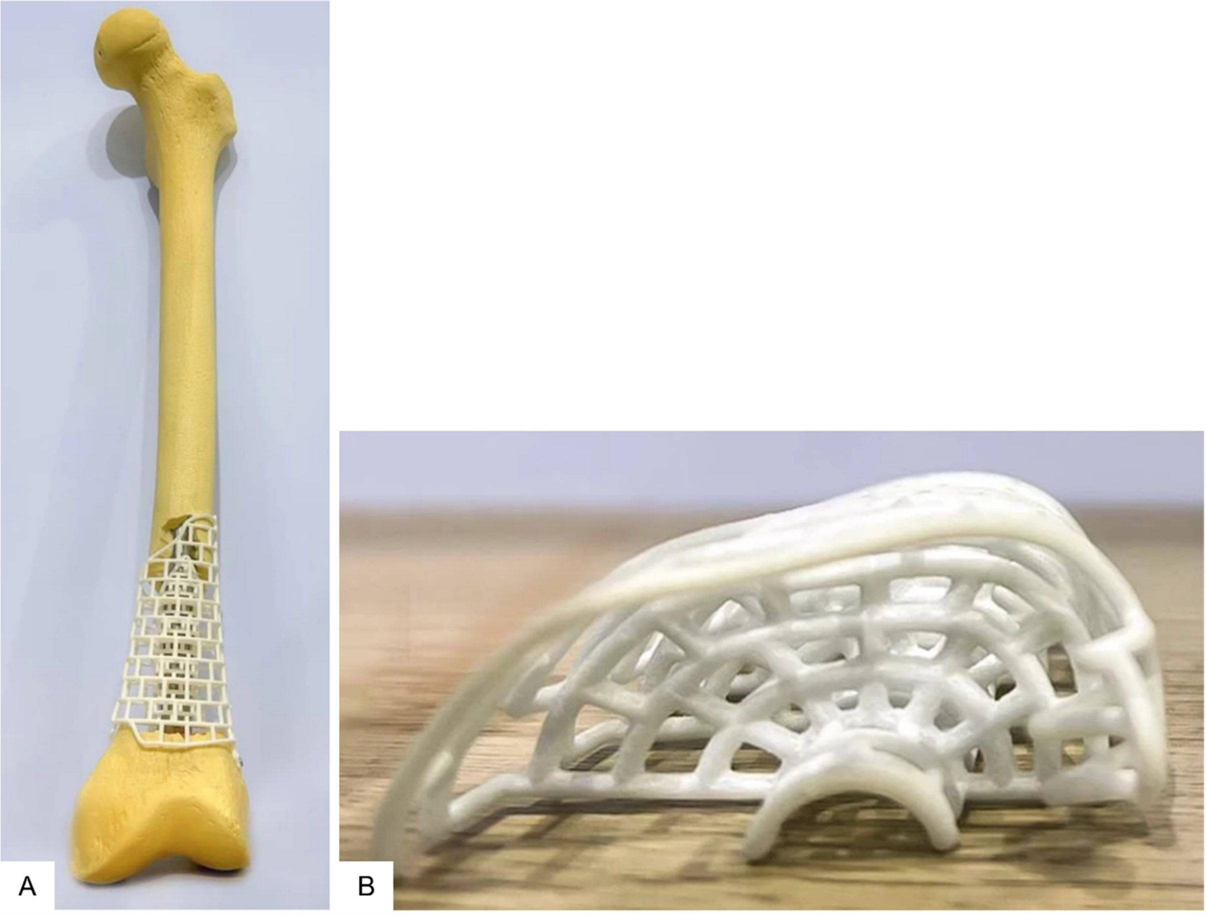

Figure S5. Annual growth of studies on scaffolds for bone tissue engineering published in PubMed between 1996 and 2023. Evaluation performed on 08.01.2023 using the RISmed package in R statistical software (version 4.0.2; R Foundation for Statistical Computing, Vienna, Austria) with the search strategy "((engineering) AND (bone)) AND (scaffold)".

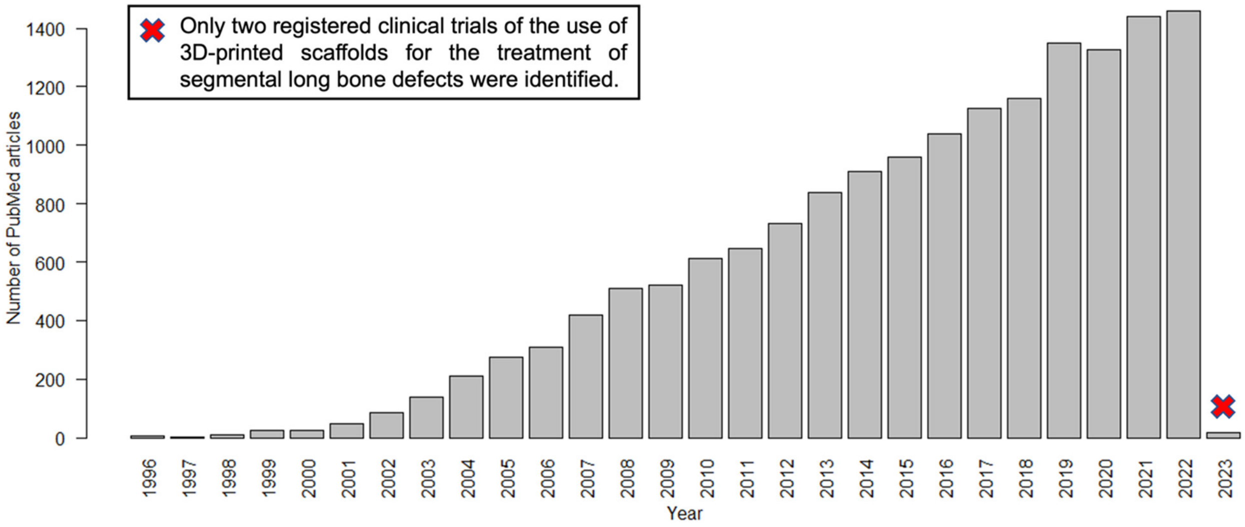

Figure S6. This 16-year-old patient, who was treated with a patient-specific Ti-6Al-4V-based implant after bone resection of an Ewing sarcoma (A), suffered a fracture at the proximal implant-bone interface (distal hole of the side plate, red circle in A) one year after surgery due to a traumatic fall. Notably, this implant failure resulted in resection of the distal femur and total knee replacement. The authors [5] decided to perform a retrospective finite element analysis (FEA) to investigate the implant failure (B). According to the FEA results, the maximum stress generated in the inferior region of the lateral plate, the region where the implant failed, had a safety factor (SF) of 0.778, less than 1, making the implant unsafe [5]. Therefore, we agree with the authors that it makes sense to include a full FEA analysis in the workflow as part of the implant design phase, especially when using titanium implants. Reprinted with permission from Ref [5].

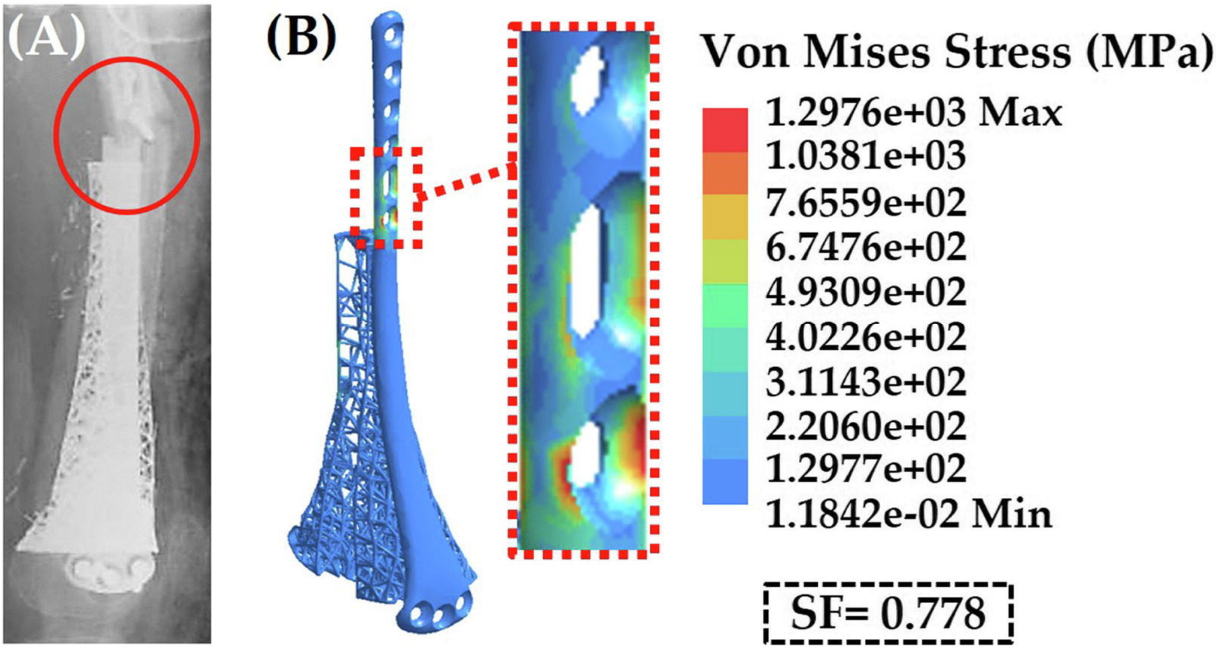

References

1. Gugala, Z.; Lindsey, R.W.; Gogolewski, S. New Approaches in the Treatment of Critical-Size Segmental Defects in Long Bones. *Macromolecular Symposia* **2007**, *253*, 147-161, doi:10.1002/masy.200750722.
2. Tetsworth, K.; Woloszyk, A.; Glatt, V. 3D printed titanium cages combined with the Masquelet technique for the reconstruction of segmental femoral defects: Preliminary clinical results and molecular analysis of the biological activity of human-induced membranes. *OTA Int* **2019**, *2*, e016, doi:10.1097/oi9.0000000000000016.
3. Nolte, P.-C.; Kemmerer, M.; Spranger, N.; Hackl, S.; von Recum, J.; Grützner, P.A.; Reiter, G. „Plate-assisted bone segment transport" bei Knochendefekten an der unteren Extremität. *Die Unfallchirurgie* **2023**, *126*, 200-207, doi:10.1007/s00113-023-01291-3.
4. Liodakis, E.; Pacha, T.O.; Aktas, G.; Sehmisch, S.; Mommsen, P. [Biological reconstruction of large bone defects : Masquelet technique and new procedures]. *Die Unfallchirurgie* **2023**, *126*, 184-189, doi:10.1007/s00113-022-01267-9.
5. Benady, A.; Meyer, S.J.; Golden, E.; Dadia, S.; Katarivas Levy, G. Patient-specific Ti-6Al-4V lattice implants for critical-sized load-bearing bone defects reconstruction. *Materials & Design* **2023**, *226*, 111605, doi:10.1016/j.matdes.2023.111605.
